# Supplementary material for: Australian Communities That Care (CTC) intervention: Benefit-cost analysis of a community-based youth alcohol prevention initiative
Source: PLoS One. 2024 Nov 27;19(11):e0314153. doi: 10.1371/journal.pone.0314153 (PMC11602060; doi:10.1371/journal.pone.0314153)
Supplement: S1 Table — (DOCX) [file pone.0314153.s001.docx]

# **S1: Duration of the Australian CTC implementation**

|  | **Cycle 1** | | **Cycle 2** | |
| --- | --- | --- | --- | --- |
| **CTC community** | **Year began cycle 1** | **Year finished cycle 1** | **Year began cycle 2** | **Year finished cycle 2** |
| Community coalition 1 | 2000 | 2005 | 2006 | 2012 |
| Community coalition 2 | 2000 | 2009 | - | - |
| Community coalition 3 | 2000 | 2006 | - | - |
| Community coalition 4 | 2009 | 2015 | - | - |
