# Supplementary material for: Australian Communities That Care (CTC) intervention: Benefit-cost analysis of a community-based youth alcohol prevention initiative
Source: PLoS One. 2024 Nov 27;19(11):e0314153. doi: 10.1371/journal.pone.0314153 (PMC11602060; doi:10.1371/journal.pone.0314153)
Supplement: S1 Fig — (DOCX) [file pone.0314153.s002.docx]

**S2 Fig: Example anonymised Australian CTC community cost template**

**Reference Links:**

Local Award file:///D:/UserData/abimanyi/Downloads/social-and-community-services-victoria-award-ap796561-pay-guide.pdf

Deakin Salaries https://www.deakin.edu.au/__data/assets/pdf_file/0018/41472/salary-rates-academic.pdf

Infaltion rates https://www.rba.gov.au/calculator/annualPreDecimal.html
