# Supplementary material for: Australian Communities That Care (CTC) intervention: Benefit-cost analysis of a community-based youth alcohol prevention initiative
Source: PLoS One. 2024 Nov 27;19(11):e0314153. doi: 10.1371/journal.pone.0314153 (PMC11602060; doi:10.1371/journal.pone.0314153)
Supplement: Attachment — Submitted filename: Comments_DR.docx [file pone.0314153.s004.docx]

PLOS ONE

Australian Communities That Care (CTC) intervention: Benefit-cost analysis of a community-based youth alcohol prevention initiative

--Manuscript Draft--

| Manuscript Number: | PONE-D-24-08327R1 |
| --- | --- |
| Article Type: | Research Article |
| Full Title: | Australian Communities That Care (CTC) intervention: Benefit-cost analysis of a community-based youth alcohol prevention initiative |
| Short Title: | Community-based 'Communities That Care (CTC) intervention: Benefit-cost analysis in the Australian setting |
| Corresponding Author: | Julie Abimanyi-Ochom Deakin University  Burwood, 3125, Vic, Australia, VIC AUSTRALIA |
| Keywords: | cost-benefit analysis, community-based intervention, Australian Communities That Care, youth alcohol prevention, CTC program Australia. |
| Abstract: | Background: Internationally, adolescent alcohol consumption has been a major community concern for decades. Globally, there is a growing array of interventions aimed at preventing youth alcohol-related problems. Notably, the Communities that Care (CTC) process in the USA has proven to be a cost-effective intervention, leading to a reduction in adolescent alcohol-related problems. In Australia, the CTC trial has shown positive outcomes such as reduced adolescent alcohol and drug use, antisocial behaviors, and the availability of alcohol in the community. To encourage wider adoption, it is essential to ascertain the cost-effectiveness of the Australian CTC trial and its potential impact on addressing youth alcohol problems.  Method: We conducted a societal perspective benefit-cost analysis focused on reducing adolescent (aged 10-14) alcohol use in the initial four CTC communities in Australia, spanning 2001 to 2015. To ensure accuracy, estimated benefits were adjusted for joint effects to prevent the double counting of the benefits over time.  Results: The Australian CTC trial, as an adolescent alcohol prevention strategy, demonstrates robust economic credentials, delivering a return of AUD 2.6 for each dollar invested. The cost savings resulting from the reduction in alcohol consumption were estimated at AUD 123 per youth per 15 years and AUD 8 per person per year. The average cost of the CTC trial amounted to AUD 48 per youth over 15 years and AUD 3 per youth per year. The largest contribution to primary benefits were reductions in crime and violence with 93% of the total benefits.  Conclusions: This study makes a valuable contribution to the international economic evidence related to youth alcohol prevention initiatives. The results affirm the strong cost-effectiveness potential of the initial CTC trial implementation in Australia.  Moreover, conducting further cost-benefit modelling for additional outcomes and beyond the intervention’s timeframe is likely to enhance the economic viability of the CTC trial. |
| Order of Authors: | Julie Abimanyi-Ochom |
|  | Sithara Wanni Arachchige Dona |
|  | Shalika Bohingamu Mudiyanselage |
|  | Kanika Mehta |
|  | Margaret Kuklinski |
|  | Bosco Rowland |
|  | John Toumbourou |
|  | Rob Carter |
| Response to Reviewers: | The response to the specific reviewer and editor comments have been included in the response to reviewers document. |
| Additional Information: |  |

| Question | Response |
| --- | --- |
| Financial Disclosure  Enter a financial disclosure statement that describes the sources of funding for the work included in this submission. Review the [submission guidelines](http://journals.plos.org/plosone/s/submission-guidelines#loc-financial-disclosure-statement) for detailed requirements. View published research articles from [*PLOS ONE*](https://journals.plos.org/plosone/) for specific examples.  This statement is required for submission and will appear in the published article if the submission is accepted. Please make sure it is accurate. | Yes |
| Funded studies  Enter a statement with the following details:   - Initials of the authors who received each award - Grant numbers awarded to each author - The full name of each funder - URL of each funder website - Did the sponsors or funders play any role in the study design, data collection and analysis, decision to publish, or preparation of the manuscript? |  |
| Did you receive funding for this work? |  |
| Please add funding details.  as follow-up to "Financial Disclosure  Enter a financial disclosure statement that describes the sources of funding for the work included in this submission. Review the [submission guidelines](http://journals.plos.org/plosone/s/submission-guidelines#loc-financial-disclosure-statement) for detailed requirements. View published research articles from [*PLOS ONE*](https://journals.plos.org/plosone/) for specific examples.  This statement is required for submission and will appear in the published article if the submission is accepted. Please make sure it is accurate. | Bosco Rowland, John W Toumbourou and Rob Carter received funding from The National Health and Medical Research Council (APP1087781) and Australian Research Council (LP100200755). |

| Funded studies  Enter a statement with the following details:   - Initials of the authors who received each award - Grant numbers awarded to each author - The full name of each funder - URL of each funder website - Did the sponsors or funders play any role in the study design, data collection and analysis, decision to publish, or preparation of the manuscript? |  |
| --- | --- |
| Did you receive funding for this work?" |  |
| Please select the country of your main research funder (please select carefully as in some cases this is used in fee calculation).  as follow-up to "Financial Disclosure  Enter a financial disclosure statement that describes the sources of funding for the work included in this submission. Review the [submission guidelines](http://journals.plos.org/plosone/s/submission-guidelines#loc-financial-disclosure-statement) for detailed requirements. View published research articles from [*PLOS ONE*](https://journals.plos.org/plosone/) for specific examples.  This statement is required for submission and will appear in the published article if the submission is accepted. Please make sure it is accurate. | AUSTRALIA - AU |
| Funded studies  Enter a statement with the following details:   - Initials of the authors who received each award - Grant numbers awarded to each author - The full name of each funder - URL of each funder website - Did the sponsors or funders play any role in the study design, data collection and analysis, decision to publish, or preparation of the manuscript? |  |
| Did you receive funding for this work?" |  |
| Competing Interests | The following authors served in the not-for-profit company Communities That Care Ltd |

| Use the instructions below to enter a competing interest statement for this submission. On behalf of all authors, disclose any [competing interests](http://journals.plos.org/plosone/s/competing-interests) that could be perceived to bias this work—acknowledging all financial support and any other relevant financial or non- financial competing interests.  This statement is required for submission and will appear in the published article if the submission is accepted. Please make sure it is accurate and that any funding sources listed in your Funding Information later in the submission form are also declared in your Financial Disclosure statement.  View published research articles from  [*PLOS ONE*](http://journals.plos.org/plosone/) for specific examples. | that was evaluated in this paper:  Rowland (current Chief Executive Officer, CEO) and Toumbourou (Director and ex- CEO). Conflict of interest management strategies were approved by their Deakin University employer. |
| --- | --- |
| NO authors have competing interests  Enter: *The authors have declared that no competing interests exist.*  Authors with competing interests  Enter competing interest details beginning with this statement:  *I have read the journal's policy and the authors of this manuscript have the following competing interests: [insert competing interests here]* |  |
| * typeset |  |
| Ethics Statement  Enter an ethics statement for this submission. This statement is required if the study involved:   - Human participants - Human specimens or tissue - Vertebrate animals or cephalopods - Vertebrate embryos or tissues | N/A  The study didn’t require ethics approval since it used secondary data sources from the CTC study team, published journal articles/reports and publicly available data to model benefits and costs associated with the CTC intervention. The study didn’t include individual human participants’ data but rather used publicly available non-identifiable data on several outcomes in the model. |

| - Field research   Write "N/A" if the submission does not require an ethics statement.  General guidance is provided below. Consult the [submission guidelines](http://journals.plos.org/plosone/s/submission-guidelines#loc-guidelines-for-specific-study-types) for detailed instructions. Make sure that all information entered here is included in the Methods section of the manuscript. |  |
| --- | --- |

| Format for specific study types  Human Subject Research (involving human participants and/or tissue)   - Give the name of the institutional review board or ethics committee that approved the study - Include the approval number and/or a statement indicating approval of this research - Indicate the form of consent obtained (written/oral) or the reason that consent was not obtained (e.g. the data were analyzed anonymously)   Animal Research (involving vertebrate animals, embryos or tissues)   - Provide the name of the Institutional Animal Care and Use Committee (IACUC) or other relevant ethics board that reviewed the study protocol, and indicate whether they approved this research or granted a formal waiver of ethical approval - Include an approval number if one was obtained - If the study involved *non-human primates*, add *additional details* about animal welfare and steps taken to ameliorate suffering - If anesthesia, euthanasia, or any kind of animal sacrifice is part of the study, include briefly which substances and/or methods were applied   Field Research  Include the following details if this study involves the collection of plant, animal, or other materials from a natural setting:   - Field permit number - Name of the institution or relevant body that granted permission |  |
| --- | --- |
|  |  |
| Data Availability  Authors are required to make all data underlying the findings described fully available, without restriction, and from the time of publication. PLOS allows rare exceptions to address legal and ethical concerns. See the [PLOS Data Policy](http://journals.plos.org/plosone/s/data-availability) and [FAQ](http://journals.plos.org/plosone/s/data-availability#loc-faqs-for-data-policy) for detailed information. | Yes - all data are fully available without restriction |

| A Data Availability Statement describing where the data can be found is required at submission. Your answers to this question constitute the Data Availability Statement and will be published in the article, if accepted. |  |
| --- | --- |
| Important: Stating ‘data available on request from the author’ is not sufficient. If your data are only available upon request, select ‘No’ for the first question and explain your exceptional situation in the text box. |  |
| Do the authors confirm that all data underlying the findings described in their manuscript are fully available without restriction? |  |
| Describe where the data may be found in full sentences. If you are copying our sample text, replace any instances of XXX with the appropriate details.   - If the data are held or will be held in a public repository, include URLs, accession numbers or DOIs. If this information will only be available after acceptance, indicate this by ticking the box below. For example: *All XXX files are available from the XXX database (accession number(s) XXX, XXX.*). - If the data are all contained within the manuscript and/or Supporting Information files, enter the following: *All relevant data are within the manuscript and its Supporting Information files.* - If neither of these applies but you are able to provide details of access elsewhere, with or without limitations, please do so. For example:   *Data cannot be shared publicly because of [XXX]. Data are available from the XXX Institutional Data Access / Ethics Committee (contact via XXX) for researchers who meet the criteria for access to confidential data.*  *The data underlying the results presented in the study are available from (include the name of the third party* | All relevant data are within the manuscript and its Supporting Information files. |

| *and contact information or URL).*   - This text is appropriate if the data are owned by a third party and authors do not have permission to share the data.   * typeset |  |
| --- | --- |
| Additional data availability information: |  |

<https://www2.cloud.editorialmanager.com/pone/download.aspx?id=37099143&guid=157d08b5-623c-42ec-8f4b-c9c9f0ca143d&scheme=1>1

#### 1 Australian Communities That Care (CTC) intervention: Benefit-cost analysis of a

2 **community-based youth alcohol prevention initiative**

##### 3 Julie Abimanyi-Ochom^1&*^, Sithara Wanni Arachchige Dona^1&^, Shalika Bohingamu Mudiyanselage^1^,

4 **Kanika Mehta^2^, Margaret Kuklinski^3^, Bosco Rowland^4&5^, John W Toumbourou^4^ and Rob Carter^1¶^**

5

6 ^1^ Deakin Health Economics, School of Health and Social Development, Institute for Health Transformation,
7 Faculty of Health, Deakin University, Geelong, Victoria, Australia
8 ^2^ School of Medicine, IMPACT – The Institute for Mental and Physical Health and Clinical Translation, Deakin
9 University, Geelong, Victoria, Australia
10 ^3^ Social Development Research Group, School of Social Work, University of Washington, Seattle, Washington,
11 USA
12 ^4^ School of Psychology, Centre for Social and Early Emotional Development, Faculty of Health, Deakin
13 University, Geelong, Victoria, Australia
14 ^5^ Eastern Health Clinical School & Monash Addiction Research Centre, Monash University, Victoria, Australia 15
16 ^¶^Joint senior authorship
17 ^&^Joint first authorship 18

19

20

21

22

23

24

25

26

27

28

29

30

31

32 **^*^Corresponding Author**
33 Julie Abimanyi-Ochom
34 Deakin Health Economics, School of Health and Social Development, Institute for Health Transformation, Faculty
35 of Health, Deakin University, Geelong, Victoria, 3220, Australia
36 Email: [j.abimanyiochom@deakin.edu.au](mailto:j.abimanyiochom@deakin.edu.au)

37

38

39

40

41	**Abstract**

42 **Background:** Internationally, adolescent alcohol consumption has been a major community
43 concern for decades. Globally, there is a growing array of interventions aimed at preventing
44 youth alcohol-related problems. Notably, the Communities that Care (CTC) process in the USA
45 has proven to be a cost-effective intervention, leading to a reduction in adolescent alcohol-
46 related problems. In Australia, the CTC trial has shown positive outcomes such as reduced
47 adolescent alcohol and drug use, antisocial behaviors, and the availability of alcohol in the
48 community. To encourage wider adoption, it is essential to ascertain the cost-effectiveness of
49 the Australian CTC trial and its potential impact on addressing youth alcohol problems.
50 **Method:** We conducted a societal perspective benefit-cost analysis focused on reducing
51 adolescent (aged 10-14) alcohol use in the initial four CTC communities in Australia, spanning
52 2001 to 2015. To ensure accuracy, estimated benefits were adjusted for joint effects to prevent
53 the double counting of the benefits over time.
54 **Results:** The Australian CTC trial, as an adolescent alcohol prevention strategy, demonstrates
55 robust economic credentials, delivering a return of AUD 2.6 for each dollar invested. The cost
56 savings resulting from the reduction in alcohol consumption were estimated at AUD 123 per
57 youth per 15 years and AUD 8 per person per year. The average cost of the CTC trial amounted
58 to AUD 48 per youth over 15 years and AUD 3 per youth per year. The largest contribution to
59 primary benefits were reductions in crime and violence with 93% of the total benefits.
60 **Conclusions:** This study makes a valuable contribution to the international economic evidence
61 related to youth alcohol prevention initiatives. The results affirm the strong cost-effectiveness
62 potential of the initial CTC trial implementation in Australia. Moreover, conducting further
63 cost-benefit modelling for additional outcomes and beyond the intervention’s timeframe is
64 likely to enhance the economic viability of the CTC trial.
65 ***Keywords:*** cost-benefit analysis, community-based intervention, Australian Communities That
66 Care, youth alcohol prevention, CTC program Australia.

# 67 Introduction

68 Globally, excessive alcohol consumption is a leading risk factor for death and disability ^1^.
69 Alcohol consumption often begins during the early teenage years, and risky levels of
70 consumption during the teen years are associated with harmful consumption in the adult years
71 ^2^. Australia has the highest rates of adolescent alcohol use in the world ^3^. If alcohol related
72 health and community issues are to be avoided, cost-effective programs and policies need to
73 be systemically implemented ^4^. Australia implemented the Communities That Care (CTC)
74 prevention framework with the aim of reducing and preventing underage alcohol consumption
75 ^4^. The CTC framework can also be described as a preventive intervention.
76 The CTC framework was originally developed in the United States (US) and was later
77 implemented as a community intervention in Australia in 2002 (trial-related prior-
78 implementation activities happened prior to 2002) ^5^, which aimed at reducing adolescent
79 alcohol and drug use by identifying and addressing local risk and protective factors ^6^.
80 CTC is designed to build prevention capacity and sustainability across large municipal
81 communities and is implemented in 5 phases ^6-8^. Communities begin by forming a municipal
82 coalition of stakeholders (Phase 1 & 2). They then collect local data and develop a profile of
83 their community (Phase 3) to identify elevated risk factors that cause adolescent alcohol use
84 (e.g., parent and community supply of alcohol, low perceived risk, poor family management).
85 Communities then use this profile to inform the selection of evidence-based strategies that
86 target elevated risk factors (e.g., programs to reduce underage alcohol supply, parent
87 education), while also increasing protective factors such as community rewards and
88 opportunities (Phase 4). Communities then implement and evaluate evidence-based strategies
89 (Phase 5), and monitor their progress by repeating the survey again, and comparing to their
90 Phase 3 results. The CTC trial was conducted in four Australian community coalitions (Local
91 Government Areas (LGA) across Australia (in Victoria and Western Australia) from 2002 to
92 2015. The evaluation compared 104 non-CTC LGAs ^6^. The CTC trial in Australia evaluated
93 data collected over a 15-year period.
94 The CTC trial action plans focused on reducing family risk factors and favorable attitudes to
95 alcohol and drugs in youth using school-based and parent/family drug education ^9,10^.
96 Supporting information S1 outlines the number of CTC cycles and the duration of the CTC
97 trial in the first four community coalitions. Community coalition 1 completed two cycles
98 compared to the other three communities, which completed only one cycle each.
99 The CTC trial in US has demonstrated reductions in adolescent alcohol and drug use, and
100 related antisocial behaviors, less favorable parent attitudes toward alcohol and drug use, and
101 reduced community availability of alcohol ^11,12^. Given these novel findings, this study aims to
102 develop a benefit-cost analysis model to explore the cost and benefits from a	societal
103 perspective (i.e., all costs and outcomes to whomever they accrue which includes health sector
104 (public/private) or governments or individuals/patients/families, or taxpayer or third party
105 funders ^13^) using data from a 15-year evaluation of the Australian CTC intervention in 4 trial
106 communities. Benefit-cost analysis will inform CTC trial effectiveness and guide policymakers
107 and stakeholders interested in achieving positive youth outcomes in a cost-effective way.
108 A benefit-cost analysis places a monetary value on significant intervention-related outcomes,
109 based on benefits expected to accrue over the reference period for the participants. The present
110 economic analysis of the Australian CTC trial seeks to determine whether the benefits
111 associated with the observed effects of the CTC trial outweigh the costs of implementation.
112 The benefit-cost analysis results provide economic evidence related to the CTC trial and return
113 on investment of public dollars, even under very conservative cost and benefit assumptions.

# 114 Material and methods

115 The benefit-cost model estimated the cost of the intervention and the benefits in the youth
116 population based on the reduction in alcohol use due to the CTC trial. Firstly, the reduction in
117 alcohol use measured in the trial was used to estimate the reduction in the alcohol consuming
118 population due to the intervention for the intervention population. Secondly, we estimated the
119 population that have risky alcohol consumption within the alcohol consuming intervention
120 population. Lastly, avoided consequences in health-related events (alcohol-related hospital
121 admissions, emergency department visits, ambulance attendances, alcohol treatment
122 programs), and in non-health events (deaths, and crime) due to risky alcohol consumption were
123 estimated and costed to give the benefit from the CTC intervention.

## 124 Study design

125

126 An intervention-based benefit-cost model was conducted from a societal perspective.

## 127 Model period

128 The periods for the benefit-cost model were derived using the Australian Bureau of Statistics
129 (ABS) data periods, which provides five-year population data estimates. ^14-16^. This provided
130 three model periods covering 15 years of the trial: Period 1; 2001-2005, Period 2; 2006-2010
131 and Period 3; 2011-2015.

## 132 Model Population

133 The ABS population numbers were aggregated by CTC trial LGAs, age, sex, and year ^14-16^.
134 The ABS five-year population numbers were combined with survey estimates of the proportion
135 of alcohol consumption ^17^ for each age group to estimate the population who do consume
136 alcohol (Figure 1).
137 [Insert Figure 1: Model population 2001-2015]

138

139

140

## 141 Model inputs

142 Through interpolation of results of Toumbourou and colleagues study, the Australian CTC trial
143 demonstrated a larger reduction in adolescents using alcohol of 28.3% compared to control
144 communities with a 12.2% reduction over 15 years ^6^. The difference in reduction in alcohol use
145 between the two groups was 16.1 percentage points, or an average absolute reduction in use of
146 1.01% per year (i.e., 1.01= 16.1/15, 5.05% per five years) ^6^. We considered a single occasion
147 of alcohol risk ^18^, which is more applicable to the model population. Details of the model inputs
148 are shown in Table 1.

149

150 [insert table 1: Model inputs]
151 Table 1: Benefit-cost model inputs

| **Model input** | **Model Value** | **Reference and notes** |
| --- | --- | --- |
| Average absolute reduction in alcohol use due to CTC trial | 1.01% per year | Toumbourou et al., 2019 ^6^ |
| Percentage of adolescents that use alcohol (10-17 years  old) | 61.8% | Kelly et al., 2015 ^17^ |
| Percentage of adolescents within the intervention  community exposed to the CTC trial | 100% | Kuklinski et al., 2012 ^19^ |
| **Alcohol risk**  Percentage of youth using alcohol at levels that risk harm: 2006-2010 | 18-19 years old  Males: 18.5%,  Females: 15.9% | 2007 AIHW estimated alcohol- related harm from a single occasion (more than 4 drinks) by age and sex  (17.2% gender aggregated rate) ^20^ |
| **Alcohol risk**  Percentage of youth using alcohol at levels that risk harm: 2011-2015 | 18-19 years old  Males: 13.1%,  Females: 15.8%  20-24 years old  Males: 13.1%,  Females: 15.8% | 2013 AIHW alcohol-related harm from a single occasion (more than 4 drinks) by age and sex (gender aggregated rate: 18-19 years old - 8.3%, 20-24 years old – 14.3%) ^21^ |
| Percentage of youth alcohol-related emergency department visits and cost per visit | 2001, 2006, 2011:  15.1%, 24.4%, 28%  $290 (2005) | 15-24 age rate used, cost ^22,23^ |

| Percentage of youth alcohol-related hospital admissions and cost per admission | 2001, 2006, 2011:  29.3%, 39.9%,  47.6%  $22,375 (2005) | 15-24 age rate used, cost ^22,23^ |
| --- | --- | --- |
| Percentage of youth alcohol related ambulance attendance and cost per attendance | 2001, 2006, 2011:  17.9%,17.9%,56.2%  $689 (2012) | 15-24 age rate used, cost ^23,24^ |
| Percentage of youth alcohol related alcohol treatment programs and cost per program, | 2001, 2006, 2011:  45.4%, 60.5%,  50.3%  $7,285 (2011) | 15-24 age rate used, cost ^23,25^ |
| Percentage of youth alcohol-related deaths and cost per life lost | 2001, 2006, 2011:  0.67%, 0.67%,  0.52%  $1,365,563 (2010) | 15-24 age rate used, cost ^23,26^ |
| Number of overall crimes (age 10-19) | 109,844 (age 10-19) | Offenders (2008-09) ^27^ |
| Percentage of offenders involved in alcohol-related incidents | 1.4% (age 10-14),  18.8 (age 15-19) and  25.4% (age 20-24) | 2007 ^28^ |
| Alcohol attributable fraction of crime | 15% | in 2008, 2010 & 2015 ^29^ |

152 Note: AIHW- Australian Institute of Health and Welfare

153

154	**Model outputs**

155

156 Model outputs are presented as follows: (1) The Australian CTC trial cost, (2) short-term cost
157 savings (i.e., benefits) from avoided events to health, and criminal system due to CTC trial,
158 and (3) Benefit-Cost ratio (BCR) as primary outcome of CTC trial.

## 159 Data Analysis

160 The cost and benefits estimation methods are presented in Table 2. First, the cost of the CTC
161 trial was calculated for each of the four CTC trial communities using CTC trial records
162 provided by the trial implementation team using a template designed by the health economics
163 investigators (See S2 Example anonymised Australian CTC community cost template). CTC
164 costs were attributed to organizing the community coalitions, intervention programs, student
165 surveys, rental space, salary, CTC training for each of the 5 phases and other costs such as
166 transport, catering resources, and report development. Activity costs (categories) were

167

estimated using an approach

of multiplying the quantity of each resource by its unit price to

168 generate a resource cost. The costs of all resources used in an activity were then summed to

26

169 give the total cost of the activity. The activity costs were summed up to give the total cost per
170 community for the intervention period. Only the first cycle was taken into account when there
171 were multiple cycles during the intervention period (see S2 Example anonymised Australian
172 CTC community cost template).
173 Second, societal benefits from averted events resulting from the reduced alcohol consumption
174 due to the CTC trial were modelled and calculated for the 15-year intervention period ^30^. The
175 model assumption was that the reduction in alcohol consumption due to the CTC trial ^6^ led to
176 the reduction in alcohol-related adverse events and associated costs during the intervention
177 period.
178 The Australian low-risk drinking guidelines recommend that adolescents should not consume
179 alcohol before the age of 18 ^31^. Thus, the model assumed that for the 10 to 17-year-old
180 population, any alcohol consumed is harmful. For the 18 to 19-year-olds and 20 to 24-year-
181 olds alcohol was assumed harmful when consumed above the NHMRC (National Health and
182 Medical Research Council) recommended levels, more than four standard drinks in a session.
183 Avoided adverse events for alcohol users were categorised as health and non-health related. As
184 shown in Table 2, the health-related avoided events include: 1) alcohol-related hospital
185 admissions, 2) emergency department (ED) visits, 3) ambulance attendances, 4) alcohol
186 treatment programs. The non-health events include: (1) deaths, and 2) crime.
187 The benefits were adjusted for joint effects using a base model of 50%, where the total benefits
188 were halved, based on previous literature^32^adjusted for possible double counting due to
189 correlations among benefits from different outcomes ^33^. The joint effect was considered to
190 correct for shared risk factors that can contribute to the same outcomes. All nominal costs and
191 benefits estimates were converted to 2020 Australian dollars (AUD) using the health price
192 index ^34,35^. The model was generated using Microsoft Excel. The impact of intangible costs
193 and benefits such as stress and quality of life were not included in the model. The total, average,
194 weighted average intervention cost, total and average benefits, and average dollars saved per
195 youth were calculated. The benefit-cost ratio was calculated as the ratio of the average dollar
196 saved per youth and average cost per youth.

197

198

199	Table 2: Cost and benefits estimation methods

| **Costs and benefits** | Measures | Estimations and assumptions | Data source | Cost and benefits estimation method | Cost and benefits estimations |
| --- | --- | --- | --- | --- | --- |
| **Intervention cost** | Total intervention | Expenditure *Categories* | CTC implementation | CTC trial cost for each | Total CTC trial cost for each |
|  | implementation cost | 1. Administration cost | team | expenditure category per ABS | expenditure category. |
|  |  | (organizing the community |  | youth aged 10-14 years who lived |  |
|  |  | coalitions intervention |  | in the study communities in 2000- | The average cost per |
|  |  | programs, student surveys) |  | 2015 | community weighted by |
|  |  | 2. Rental space cost |  |  | community size (weighted |
|  |  | 3. Staff salary |  |  | average), which reduces the |
|  |  | 4. CTC training for each of the |  |  | impact of population size on |
|  |  | five phases |  |  | average cost for small |
|  |  | 5. Transport |  |  | population communities. |
|  |  | 6. Catering resources |  |  |  |
|  |  | 7. Report development |  |  |  |
|  |  |  |  |  |  |
| **Benefits of the** |  |  |  |  |  |
| **intervention** |  |  |  |  |  |
| **Avoided health** |  |  |  |  |  |
| **system events** |  |  |  |  |  |
| **alcohol-related Emergency** | 1. Rate of avoided youth  alcohol cases, 5.05% for |  | Toumbourou et al.,  2019 ^6^ | The total population in the CTC  communities x 5.05% | Total number of avoided  youth alcohol cases in the |
| **Department (ED) presentations** | five years  2. Percentage of alcohol- | The proportion of alcohol- | Turning point ^23^ | (Total number of avoided youth | CTC communities |
|  | attributed injuries at EDs, | attributed injuries presented at |  | cases in the CTC communities | Total number of avoided |
|  | 15.1%, 24.4% & 28.0% | EDs in 2001, 2006 & 2011 |  | (5.05%/5-years) x the proportion | alcohol-attributed ED |
|  |  |  |  | of alcohol-attributed injuries | presentations |
|  |  |  |  | presented at EDs per 10,000 |  |
|  |  |  |  | population) |  |
|  | 2. Cost per ED presentation, | Average ED treatment costs for | Turning point ^23^ | Total number of avoided alcohol- | The total cost saved from |
|  | $ 254 | all states and territories, |  | attributed ED presentations x $254 | avoided alcohol-attributed ED |
|  |  | considering both acute and |  |  | presentations |

non-acute ED expenses

**Costs and benefits**

Measures

Estimations and assumptions

Data source

Cost and benefits estimation method

Cost and benefits estimations

##### alcohol-related hospital admissions

1. Rate of avoided youth

alcohol cases, 5.05% for five years

Toumbourou et al.,

2019 ^6^

The total population in the CTC

communities x 5.05%

Total number of avoided

youth cases in the CTC communities

2. Percentage of alcohol-

attributed hospital admissions, 29.3%, 39.9%

& 47.6%

The proportion of alcohol-

attributed hospital admissions Eds in 2001, 2006 & 2011

Turning point ^23^	(Total number of avoided youth

cases in the CTC communities x the proportion of alcohol- attributed hospital admissions per 10,000 population)

Total number of avoided alcohol-attributed hospital admissions

2. Cost per hospital admission, $ 22,357

Average hospital admission treatment cost

Laslett et al., 2010 ^22^	Total number of avoided alcohol-

attributed hospital admissions x

$22,357

The total cost saved from avoided alcohol-attributed hospital admissions

##### alcohol-related ambulance attendances

1. Rate of avoided youth alcohol cases, 5.05% for five years

Toumbourou et al., 2019 ^6^

The total population in the CTC communities x 5.05%

Total number of avoided youth cases in the CTC communities

2. Percentage of alcohol- attributed ambulance attendance, 17.9%, 17.9% &

56.2%

The proportion of alcohol- attributed ambulance attendance in 2001, 2006 &

2011

Turning point ^23^	(Total number of avoided youth

cases in the CTC communities x the proportion of alcohol- attributed ambulance attendance per 10,000 population)

Total number of avoided alcohol-attributed ambulance attendance

2. Cost per ambulance attendance, $ 689

Average ambulance attendance cost

Watts et al., 2013 ^24^	Total number of avoided alcohol-

attributed hospital admissions x

$689

The total cost saved from avoided alcohol-attributed ambulance attendance

##### alcohol treatment programs

1. Rate of avoided youth alcohol cases, 5.05% for five years

Toumbourou et al., 2019 ^6^

The total population in the CTC communities x 5.05%

Total number of avoided youth cases in the CTC communities

2. Attendance percentage for alcohol-attributed treatment programs 45.4%, 60.5% &

50.3%

Participation proportion for alcohol-attributed treatment programs in 2001, 2006 &

2011

Turning point ^23^	(Total number of avoided youth

cases in the CTC communities x the participation proportion for alcohol-attributed treatment programs per 10,000 population)

Total number of avoided participations in alcohol- attributed treatment programs

**Costs and benefits**

Measures

Estimations and assumptions

Data source

Cost and benefits estimation method

Cost and benefits estimations

|  | 1. Treatment cost per case, | The average treatment cost per | Smith et al., 2014 ^25^ | Total number of avoided | The total cost saved from |
| --- | --- | --- | --- | --- | --- |
|  | $7285 | case: residential drug |  | participations in alcohol-attributed | avoided participants in |
|  |  | treatment, non-residential |  | treatment programs x $7285 | alcohol-attributed treatment |
|  |  | treatment and |  |  | programs. |
|  |  | pharmacotherapeutic |  |  |  |
|  |  | treatments |  |  |  |
| **Avoided non-health** |  |  |  |  |  |
| **system events** |  |  |  |  |  |
| **overall crimes** | 1. Rate of avoided alcohol  consumption, 5.05% for five |  | Toumbourou et al.,  2019 ^6^ | The total population in the CTC  communities x 5.05% | Total number of avoided  youth cases in the CTC |
|  | years  2. % of offenders by age |  | Palk et al., 2007 ^28^ | Total number of avoided youth | communities  Total number of avoided |
|  | involved in alcohol- |  |  | cases in the CTC communities x | crimes |
|  | attributed incidents  3. Alcohol attributable crime | Alcohol attributable crime cost | Collins et al., 2008 ^29^ | % of offenders by age  Alcohol attributable crime cost in | The total cost saved from |
|  | cost per person, $28,603.66 | in 2004-05 |  | 2004-05 x total number of avoided | avoided crimes |
|  |  |  |  | crimes |  |
| **alcohol-related deaths** | 1. Rate of avoided alcohol consumption, 5.05% for five |  | Toumbourou et al., 2019 ^6^ | The total population in the CTC communities x 5.05% | Total number of avoided youth cases in the CTC |
|  | years  2. Percentage of alcohol- | The proportion of alcohol- | Turning point ^23^ | (Total number of avoided youth | communities |
|  | attributed deaths, 0.67%, | attributed deaths in 2001, 2006 |  | cases in the CTC communities x | Total number of avoided |
|  | 0.67% & 0.52% | & 2011 |  | the proportion of alcohol- | alcohol-attributed deaths |
|  |  |  |  | attributed deaths per 10,000 |  |
|  |  |  |  | population) |  |
|  | 2. Cost per death, | Average cost per incident | Manning et al., 2013 | Total number of avoided alcohol- | The total cost saved from |

200

201

202

$1,367,230

(reduced workforce and	^26^

reduced household labor from premature mortality)

attributed deaths x $1,367,230

avoided alcohol-attributed deaths

13

## 203 Sensitivity analysis

204 A sensitivity analysis was undertaken to deal with uncertainty in average absolute reduction
205 due to the CTC trial to ensure the reliability of the BCR results and conclusions. Bootstrapping
206 was used for sensitivity analysis for the model variables. The ‘average absolute reduction’ of
207 1.01% per annum was modelled to account for fading intervention effects with ± 5 and 10%
208 variation. The variation of the proportion of youths exposed to the intervention was ± 5 and
209 10% of the base model. Similarly, the variation of other benefits variables was also applied
210 through the sensitivity analysis.

211	**Results**

212 The total youth population aged 10-14 in the four community coalitions assumed to be
213 impacted during the intervention period from 2001 to 2015 was 48,210.

214

215	**CTC costs**

216	The total cost of implementing the Australia CTC trial for the four pioneer community

217	coalitions over 15 years was estimated at AUD 2.3 million (Table 3). The majority of total

218	intervention costs were attributed to staff salary allocations (42%). Considering only the first

219	cycle for all included community coalitions, community coalition 2 had the highest intervention

220	cost estimated at AUD 0.70 million, and community coalition 4 had the lowest intervention

221	cost, estimated at AUD 0.42 million. The CTC trial cost per youth was AUD 48 for the entire

222	intervention period and AUD 3 per year per youth.

223

224	[Insert Table 3]

14

225

226

227	*Table 3: CTC trial costs by cost category (AUD2020)*

228

|  | **Duration of CTC (Years)** | **Cost categories AUD2020** | | | | | | | **Population** | **Total CTC trial cost** | **Total cost per youth** | **Total cost per year per youth** |
| --- | --- | --- | --- | --- | --- | --- | --- | --- | --- | --- | --- | --- |
|  |  | **Community mobilization cost** | **CTC trial program cost** | **Student survey cost** | **Rental space cost** | **Salary allocation costs** | **CTC**  **training cost** | **Other costs including transport** |  |  |  |  |
| Community coalition 1 | 6 | 173,892 | 17,602 | 49,724 | 7,661 | 201,755 | 1574,33 | - | 28,003 | 608,067 | **8.74** | **0.58** |
| Community coalition 2 | 13 | 121,432 | 15,626 | 88,249 | 8,616 | 280,826 | 186,802 | - | 12,199 | **704,799** | 12.61 | 0.79 |
| Community coalition 3 | 10 | 89,387 | 15,626 | 65,058 | 5,607 | 270,593 | 127,265 | 5,949 | 4,409 | 579,484 | **14.62** | 0.79 |
| Community coalition 4 | 7 | 46,066 | 36,096 | 27,153 | 7,421 | 220,770 | 83,680 | - | 3,599 | **421,187** | 12.02 | **1.12** |
| **Total** |  | 430,777 | 84,950 | 230,184 | 29,305 | 973,944 | 555,180 | 5,949 | 48,210 | 2,313,537 | 48 | 3 |
| **Proportion of total CTC trial cost** |  | 19% | 4% | 10% | 1% | 42% | 24% | 0.3% | - | - | - | - |

229 Note: Costs are reported only for the first cycle when there are two cycles (community coalition 1). Bold figures represent the highest and lowest costs. The intervention costs
230 were provided by the CTC trial team (based on actual expenses during the trial period, (considering the unit costs of each cost category for each community coalition)). The
231 intervention costs accounted for all the costs relevant to delivering the intervention including on-costs.

15

232 Regarding the CTC trial cost per youth, community coalition 1 had the lowest average
233 intervention cost, with AUD 8.74 per youth, while community coalition 3 had the highest cost,
234 at AUD 14.62 per youth. In terms of the cost per youth per year for the CTC trial, community
235 coalition 1 had the lowest average intervention cost, at AUD 0.58 per youth per year, while
236 community coalition 4 had the highest annual cost, at AUD 1.25 per youth.

## 237 CTC overall benefits

238 The avoided cost from alcohol-related events resulting from the CTC trial was estimated at
239 AUD 5.9 million (as detailed in Table 4) over the 15-year period from 2001-2015. Notably, the
240 highest avoided cost was attributed to alcohol-related crimes at AUD 5.5 million (93%), while
241 the lowest was associated with ED visits, totaling AUD 1,360 (< 1%). On average, the benefit
242 (avoided cost), per youth was AUD 123 for the entire intervention period and AUD 8 per year.
243 [Insert Table 4]

16

244 *Table 4: CTC trial benefits (avoided costs) (AUD2020) (adjusted for joint effects)*

| **CTC trial benefits (avoided costs due to alcohol consumption reduction)** | **Total benefit (AUD)** | **Benefit percentage of total benefits** | **Total benefit per youth**  **for the intervention period (AUD)** | **Benefit per**  **youth per year (AUD)** |
| --- | --- | --- | --- | --- |
| **Health-related benefits** | 281,470 | 4.8% | 5.8 | <1 |
| Alcohol-related hospital admissions | 204,747 | 3.5% | 4.2 | <1 |
| Alcohol-related ED visits | 1,360 | <1% | <1 | <1 |
| Alcohol-related ambulance attendance | 3,905 | <1% | <1 | <1 |
| Alcohol-related treatment programs | 71,458 | 1.2% | 1.5 | <1 |
| **Non-health related benefits** | 5,638,549 | 95.3% | 117 | 7.8 |
| Alcohol-related deaths (Mortality) | 157,767 | 2.7% | 3.3 | <1 |
| Alcohol-related all crimes | 5,480,782 | 92.6% | 113.7 | 7.6 |
| **Total benefits** | 5,920,018 | 100% | 123 | 8 |

245

246

247 Ninety-five percent of benefits were from non-health related avoided costs, while the other 5%
248 were from health-related avoided costs. According to the estimated total cost and total benefits,
249 the benefit cost ratio is 2.6, which translates to a return of AUD 2.6 for each dollar invested.

## 250 Sensitivity analysis

251 The sensitivity analysis found the model outputs to be robust. The BCR marginally differed for
252 each variable with ± 5 and ±10 variations. The lowest BCR was estimated at 2.34 when the
253 average absolute reduction was assumed to -10% of 1.01, while the highest BCR was at 2.86
254 when the average absolute reduction was assumed to +10% of 1.01.

255	**Discussion**

256 This study reports the benefits and cost of the inaugural Australian CTC trial undertaken with
257 4 CTC trial communities, revealing that each dollar spent on the CTC trial per youth per year
258 resulted in an estimated savings of AUD 2.6, primarily attributed to avoided alcohol-related
259 crime events. These findings offer valuable insights into quantifying and monetizing the
260 benefits of community-wide adolescent alcohol prevention initiatives within the Australian
261 context. They underscore the potential of the CTC prevention framework to improve adolescent
262 health and behavioral outcomes in Australia.
263 Intervention costs are lower in Australia, compared to the US ^19^. The Australian CTC trial cost
264 was AUD 3 per youth per year compared to AUD 199 per youth per year for the US CTC trial
265 (2004 USD 513 per youth for 5 years/ 102 per youth per year). The main reason for a lower
266 cost in the Australia CTC was that community coalition service delivery budgets were
267 redeployed in Australia compared to the US where new budgets were introduced for the CTC
268 trials. In Australia there was also a greater use of community alcohol supply reduction
269 interventions (e.g., underage test purchasing, retailer warning letters and media releases) which
270 were less costly than the family and school interventions used in the US. It is not entirely
271 comparable to contrast the US return on investment with the finding of return of investment
272 for this Australian study. ~~T.~~ The return for the US CTC trial was AUD 10.3 (2004 USD 5.3)
273 for each dollar spent compared to a return of AUD 2.6 for each dollar spent in the Australian
274 CTC trial ^19^. However, the US analysis included both the long (lifetime) and short term
275 (limiting to intervention period) harms prevented from tobacco use, delinquency, and school
276 dropout ^36^.
277 The Australian CTC trial demonstrated significant prevention effects on adolescent lifetime
278 alcohol, tobacco, and cannabis use and past year antisocial behavior [10]. However, the current
279 BCR analysis was conservative as it only included impacts associated with reduced alcohol
280 consumption over the intervention period of 15 years. Importantly, this analysis demonstrates
281 that preventive interventions can incur significant savings related to youth alcohol use over 15
282 years. Other Australian studies examining the benefit-cost of reducing alcohol consumption
283 from a societal perspective have identified larger life-time cost savings as alcohol causes high
284 costs to the society ^29,36^. However, these studies have examined adult populations and included
285 both long- and short-term economic impacts at the population level ^29^. Given that our study
286 only examined benefits during the intervention period using community level data, modelling
287 lifetime benefits may identify greater benefit-cost savings for Australian communities, as
288 evidenced from the US CTC evaluation ^19^.

289

290 The major component of costs for implementing CTC in this trial was related to staff and
291 training. This training focused on developing stakeholder knowledge of prevention science and
292 teaching them how to collect and critically interpret appropriate prevention data. The data
293 helped them strategically and systematically prioritize behavioral outcomes and recognize and
294 implement tested effective programs ^37^. As the CTC trial is also designed to build community
295 capacity, the costs needed to implement the CTC trial will likely diminish over time. In the
296 subsequent CTC cycles, communities will require less training to progress through the CTC
297 phases or will be able to facilitate their own training with minimal support. As an example, in
298 the second cycle for the community coalition community with two cycles, the cost was reduced
299 by AUD 65,736.

300

301 Most community coalition alcohol prevention programs were funded by redeploying or
302 reallocation of existing resources. Therefore, the opportunity costs of the Australian CTC trial
303 implementation were lower compared to the few communities that implemented utterly new
304 programs. This was particularly important for the feasibility of the CTC trial implementation
305 in small communities with smaller populations and fewer resources (the sample ranged from
306 3,599 to 28,003 in the four community coalition communities). This suggests that return on
307 investment for implementing the CTC framework can be partly linked with assisting
308 community investment to reorient health and evidence-based prevention services to meet
309 community needs more efficiently, rather than implementing and developing entirely new
310 services. It suggests that mobilizing communities to work within a prevention framework to
311 implement evidence-based programs or reorient services can be cost-beneficial. The
312 implementation of CTC in Australia is mainly through the re-allocation of existing resources,
313 which should help with the sustainability of CTC over time. A call for communities to reorient
314 health services has been advocated since the launch of the Ottawa Charter for Health
315 Promotion, in 1986 ^38^.
316 Other community prevention interventions, which used experimental designs, have also
317 demonstrated efficacy in preventing adolescent alcohol use ^39^. Like CTC, these interventions
318 depended on mobilizing communities and long-term prevention investment. Thus, the findings
319 of our study suggest that further examination of the economic benefits of CTC using an
320 experimental design would be a worthy investment. If possible, it would be essential to
321 compare the average absolute reduction of the BCR for different community approaches and
322 identify whether a particular approach is more effective and efficient. Based on the model
323 results, the CTC trial had a positive impact on crime due to reduction in alcohol consumption
324 among youths and therefore continuation of these kinds of interventions could significantly
325 reduce crime.

326

327 A strength of the study is its naturalistic evaluation context and, therefore, its high ecological
328 validity. It used data collected over a 15-year period to evaluate trends that are influenced by
329 real life factors and community demands. Moreover, the sensitivity analysis demonstrated the
330 results are robust with marginal changes in BCR, with the Australian CTC trial still
331 worthwhile (cost beneficial overall) after adjustment of the different rates used in the benefits
332 model.

333

## 334 Limitations and implications

335 The Australian CTC trial study was conducted as a pragmatic community coalition trial, with
336 4 trial communities, and these communities were not randomized. Data sources used did not
337 account for differences in rates of alcohol consumption and alcohol-related events by state (e.g.
338 Victoria and Western Australia) ^40^. Thus, the model may have underestimated CTC trial
339 benefits for Western Australian communities, since alcohol consumption is generally higher in
340 these communities ^41^.

341

342 Alcohol attributable event rates by age and year of occurrence were used to estimate benefits
343 from reduced ED, hospital admission, ambulance attendance, treatment programs, and death.
344 It is possible that using the same rate by gender may have overestimated benefits for females
345 and underestimated benefits for males. Furthermore, rates on ambulance attendance used
346 2006/07 rates for missing 2001 values, and rates on death used 2001 rates for missing 2006
347 rates, which may have underestimated and overestimated benefits respectively.
348 Alcohol consumption rates vary by gender and age, but this was not accounted for based on
349 data available on the CTC trial average absolute reductions in the reference sources^6^. Using an
350 average absolute reduction may have overestimated or underestimated the average absolute
351 reduction for females or males, respectively. The annualized rate of the average absolute
352 reduction over the intervention period does not account for the fading effect of the intervention.
353 However, sensitivity analysis explored uncertainty around the average absolute reduction, and
354 the BCR was still greater than 1, hence even with a possible fading intervention effect, the CTC
355 trial was still judged to be beneficial.

356

357 Similarly, the alcohol attribution fraction of crime was consistent across the period, which
358 probably led to underestimating the benefits. The measured police-reported crime prevention
359 benefit in the relevant Australian CTC evaluation 42 is consistent with that attributed in the
360 current analysis. The national rates of alcohol-related harm may not be specific to the CTC
361 communities. Rates associated with alcohol-related events may include harm due to youth
362 alcohol use and harms to youths due to other alcohol users. The model adjusted for joint effects,
363 by multiplying benefits by a factor of 0.5 using ^32^. However, there is limited evidence on
364 alternative methods to adjust for joint effects. The model input- rates for our study were based
365 on the literature (except for alcohol reduction rates). Various benefits typically included in
366 similar analyses, especially for long term or lifetime impacts, were not included in this analysis.
367 This included factors such as effects on school dropouts, employment and intangible benefits.
368 Therefore, future research on the Australian CTC should aim to estimate long term or lifetime
369 benefits by incorporating these benefits.

370	**Conclusion**

371 The benefit-cost ratio of the Australian CTC trial was found to be 2.6, which demonstrates that
372 benefits (avoided alcohol-related costs) outweighed the Australian CTC trial cost across 15
373 years. The study suggests that the CTC trial implementation is worthwhile. Findings also
374 suggest that CTC offers a cost-beneficial evidence-based approach for use by coalition
375 communities, governments, and large funding bodies to bring about population change in
376 alcohol-related health and social outcomes. This paper extends the range of cost-benefit
377 interventions available in the international context to prevent adolescent health and social
378 problems. Future research should incorporate the economic benefits of prevented youth
379 tobacco and cannabis use and antisocial behavior demonstrated in the Australian CTC Trial ^6^
380 and incorporate more robust experimental designs, average absolute reductions that accounts
381 for age, gender, length of intervention period, and long-term benefits.

382

## 383 Conflict of Interest: None

384

385

# 386 Supporting information

387

388 S1: Duration of the Australian CTC implementation
389 S2: Example anonymised Australian CTC community cost template

390

391	**References**

392

393 1.	Griswold MG, Fullman N, Hawley C, et al. Alcohol use and burden for 195 countries and territories,
394 1990–2016: a systematic analysis for the Global Burden of Disease Study 2016. The Lancet.
395 2018;392(10152):1015-1035.
396 2.	McCambridge J, McAlaney J, Rowe R. Adult consequences of late adolescent alcohol consumption: a
397 systematic review of cohort studies. PLoS medicine. 2011;8(2):e1000413.
398 3.	World Health Organization. Global status report on alcohol and health 2018,. 2019.
399 [https://www.who.int/publications/i/item/9789241565639.](https://www.who.int/publications/i/item/9789241565639) Accessed 15 August 2023.
400 4.	Patton GC, Sawyer SM, Santelli JS, et al. Our future: a Lancet commission on adolescent health and
401 wellbeing. The Lancet. 2016;387(10036):2423-2478.
402 5.	Toumbourou JW. Implementing Communities That Care in Australia: a community mobilisation
403 approach to crime prevention. Trends and issues in crime and criminal justice. 1999(122):1-6.
404 6.	Toumbourou JW, Rowland B, Williams J, Smith R, Patton G. Community intervention to prevent
405 adolescent health behavior problems: evaluation of Communities that Care in Australia. . Health Psychology.
406 2019.
407 7.	Communities That Care. 5 Phases of CTC. 2023. [https://www.communitiesthatcare.org.au/5-phases-ctc.](https://www.communitiesthatcare.org.au/5-phases-ctc)
408 Accessed 10 November 2023.
409 8.	Fagan AA, Hawkins JD, Catalano RF. Communities that care: Building community engagement and
410 capacity to prevent youth behavior problems: Oxford University Press, USA; 2018.
411 9.	McBride NT, Farringdon FH, Kennedy CA. Research to practice—formal dissemination of the School
412 Health and Alcohol Harm Reduction Project (SHAHRP) in Australia. Drug and Alcohol Review. 2007;26(6):665-
413 672.
414 10.	Sanders MR, Kirby JN, Tellegen CL, Day JJ. The Triple P-Positive Parenting Program: A systematic
415 review and meta-analysis of a multi-level system of parenting support. Clinical psychology review.
416 2014;34(4):337-357.
417 11.	Hawkins J, Oesterle S, Brown EC, et al. Results of a type 2 translational research trial to prevent
418 adolescent drug use and delinquency: A test of communities that care. Archives of Pediatrics & Adolescent
419 Medicine. 2009;163(9):789-798.
420 12.	Hawkins J, Oesterle S, Brown EC, et al. Sustained decreases in risk exposure and youth problem
421 behaviors after installation of the communities that care prevention system in a randomized trial. Archives of
422 Pediatrics & Adolescent Medicine. 2012;166(2):141-148.
423 13.	Drummond MF, Sculpher MJ, Claxton K, Stoddart GL, Torrance GW. Methods for the economic
424 evaluation of health care programmes: Oxford university press; 2015.
425 14.	Population by Age and Sex, Australia 2006 [Internet]. 2007 [cited February 2017].
426 15.	Australian Bureau Statistics. 3235.0 Population by Age and Sex, Regions of Australia Canberra:
427 Australian	Bureau	of	Statistics	2012	[updated	Februaury	2017.
428 2012:[[http://www.abs.gov.au/AUSSTATS/abs@.nsf/DetailsPage/3235.02011?OpenDocument.](http://www.abs.gov.au/AUSSTATS/abs%40.nsf/DetailsPage/3235.02011?OpenDocument)	Accessed	10
429 January 2021.
430 16.	Australian Bureau of Statistics. Estimated Resident Population : ERP by LGA (ASGS 2013), Age and
431 Sex, 2001 to 2013. 2013. Data extracted on 13 Apr 2017 06:44 UTC (GMT) from ABS.Stat © Commonwealth of
432 Australia. Creative Commons Attribution 2.5 Australia (<https://creativecommons.org/licenses/by/2.5/au>).
433 Accessed April 2017.
434 17.	Kelly AB, Evans‐Whipp TJ, Smith R, et al. A longitudinal study of the association of adolescent polydrug
435 use, alcohol use and high school non‐completion. Addiction 2015;110:627-635.
436 18.	Degenhardt L, O'Loughlin C, Swift W, et al. The persistence of adolescent binge drinking into adulthood:
437 findings from a 15-year prospective cohort study. BMJ open. 2013;3(8):e003015.
438 19.	Kuklinski MR, Briney JS, Hawkins JD, Catalano RF. Cost-benefit analysis of communities that care
439 outcomes at eighth grade. Prevention Science. 2012;13(2):150-161.
440 20.	Australian Institute of healtha nd Welfare. 2010 National Drug Strategy Household Survey report.
441 Australian Institute of Health and Welfare; 2011.
442 21.	Health AIo, Welfare. National Drug Strategy Household Survey detailed report: 2013. Canberra: AIHW;
443 2014.
444 22.	Laslett A, Catalano P, Chikritzhs T, et al. The range and magnitude of alcohol’s harm to others. Fitzroy:
445 AER Centre for Alcohol Policy Research, Turning Point Alcohol and Drug Centre, Eastern Health; 2010.
446 23.	Turning	Point.	AODstats	-	Victoria	State.	Retrieved	Februaury,	2017,	from
447 <http://aodstats.org.au/VicState/> 2017. [http://aodstats.org.au/VicState/.](http://aodstats.org.au/VicState/) Accessed Februaury, 2017.
448 24.	Watts JJ, Abimanyi-Ochom J, Sanders KM. Osteoporosis costing all Australian: a new burden of disease
449 analysis-2012 to 2022. 2013.
450 25.	Smith RG, Jorna P, Sweeney J, Fuller G. Counting the costs of crime in Australia: A 2011 estimate. AIC
451 reports Research and Public Policy series. 2014:xvii.
452 26.	Manning M, Smith C, Mazerolle P. The societal costs of alcohol misuse in Australia. Trends and issues
453 in crime and criminal justice. 2013(454):1-6.
454 27.	Australian Bureau of Statistics. Recorded Crime Offenders 'Offenders, Australia' [data set]. 2022.
455 <https://www.abs.gov.au/statistics/people/crime-and-justice/recorded-crime-offenders/latest-release#content>
456 Accessed 10 March 2022.
457 28.	Palk G, Davey J, Freeman J. Prevalence and characteristics of alcohol-related incidents requiring police
458 attendance. Journal of Studies on Alcohol and Drugs. 2007;68(4):575-581.
459 29.	Collins D, Lapsley HM. The costs of tobacco, alcohol and illicit drug abuse to Australian society in
460 2004/05: Department of Health and Ageing Canberra; 2008.
461 30.	McCollister KE, French MT, Fang H. The cost of crime to society: new crime-specific estimates for
462 policy and program evaluation Drug and Alcohol Dependence. 2010;108::98–109.
463 31.	NHMRC. Australian guidelines to reduce health risks from drinking alcohol. Canberra, ACT: National
464 Health and Medical Research Council; 2009.
465 32.	Cadilhac D, Magnus A, Cumming T, et al. The health and economic benefits of reducing disease risk
466 factors. Prepared for VicHealth by Deakin Health Economics Group, Deakin University and National Stroke
467 Research Institute
468 2009.
469 33.	Vos T, Carter R, Barendregt J, et al. Assessing cost-effectiveness in prevention. The University of
470 Queensland, Brisbane, and Deakin University, Melbourne. 2010.
471 34.	Australian Institute of healtha nd Welfare. Annal rates of health inflation, 1999-00 to 2009-19. 2011
472 [http://www.aihw.gov.au/publication-detail/?id=10737420435.](http://www.aihw.gov.au/publication-detail/?id=10737420435) Accessed February 20 2022.
473 35.	Reserve	Bank	of	Australia.	Infaltion	calculator.	Reserve	Bank	of	Australia.
474 [https://www.rba.gov.au/calculator/annualDecimal.html.](https://www.rba.gov.au/calculator/annualDecimal.html) Accessed April 26, 2020 2022.
475 36.	Miller TR, Levy DT, Spicer RS, Taylor DM. Societal costs of underage drinking. Journal of studies on
476 alcohol. 2006;67(4):519-528.
477 37.	Communities That Care. A guide to Australian Prevention Strategies. Victoria: Communities That Care
478 Ltda. 2012.
479 38.	Charter O. Ottawa Charter for health promotion. First international conference on health promotion.
480 1986;21:17-21.
481 39.	Perry CL, Williams CL, Veblen-Mortenson S, et al. Project northland: Outcomes from a communitywide
482 alcohol use prevention program during early adolescence. American Journal of Public Health. 1996;86(7):956 -
483 965.
484 40.	Toumbourou JW, Rowland B, Matin G-M, Patton G, Williams J. Student survey trends in reported
485 alcohol use and influencing factors in Australia. Drug and Alcohol Review. 2018.
486 41.	Australian Bureau of Statistics. 4364.0.55.001 - National Health Survey: First Results, 2014-2015.
487 Canberra:	Australian	Bureau	of	Statistics	2015	[updated	August	2019.	December
488 2015:[[https://www.abs.gov.au/ausstats/abs@.nsf/Lookup/by%20Subject/4364.0.55.001~2014-](https://www.abs.gov.au/ausstats/abs%40.nsf/Lookup/by%20Subject/4364.0.55.001~2014-15~Main%20Features~Alcohol%20consumption~25)
489 [15~Main%20Features~Alcohol%20consumption~25.](https://www.abs.gov.au/ausstats/abs%40.nsf/Lookup/by%20Subject/4364.0.55.001~2014-15~Main%20Features~Alcohol%20consumption~25) Accessed 10 January 2021.
490 42.	Rowland B, Kelly AB, Mohebbi M, et al. Evaluation of communities that care—Effects on municipal
491 youth crime rates in Victoria, Australia: 2010–2019. Prevention science. 2022:1-12.

492

Figure	[Click here to access/download;Figure;Figure 1.docx](https://www2.cloud.editorialmanager.com/pone/download.aspx?id=37099150&guid=dfbfbfc5-c2ea-434a-9845-3d14a3bd6d8e&scheme=1)[<https://www2.cloud.editorialmanager.com/pone/download.aspx?id=37099150&guid=dfbfbfc5-c2ea-434a-9845-3d14a3bd6d8e&scheme=1>](https://www2.cloud.editorialmanager.com/pone/download.aspx?id=37099150&guid=dfbfbfc5-c2ea-434a-9845-3d14a3bd6d8e&scheme=1)

Figure 1: Model population 2001-2015

| Period 1:  2001-2005 | 10–14-year-old ABS population x % of  population who consumed alcohol |  | |
| --- | --- | --- | --- |
| Period 2:  2006-2010 | 10–14-year-old ABS population (new population in Period 2) x % of population who consumed alcohol | 15–19-year-old ABS population (Period 1 population continues to Period 2) x % of  population who consumed alcohol |  |
| Period 3:  2011-2015 | 10–14-year-old ABS population (new population in Period 3) x % of population who consumed alcohol | 15–19-year-old ABS population (Period 2 population continues to Period 3) x % of  population who consumed alcohol | 20–24-year-old ABS population (Period 1 population continues to Period 3) x % of  population who consumed alcohol |

Note: x indicates multiplication

[Click here to access/download](https://www2.cloud.editorialmanager.com/pone/download.aspx?id=37099151&guid=3a513a80-659a-422f-9055-5ede03630adf&scheme=1)

[Supporting Information](https://www2.cloud.editorialmanager.com/pone/download.aspx?id=37099151&guid=3a513a80-659a-422f-9055-5ede03630adf&scheme=1)

[S1 Table_Duration of the Australian CTC](https://www2.cloud.editorialmanager.com/pone/download.aspx?id=37099151&guid=3a513a80-659a-422f-9055-5ede03630adf&scheme=1)

[implementation.docx](https://www2.cloud.editorialmanager.com/pone/download.aspx?id=37099151&guid=3a513a80-659a-422f-9055-5ede03630adf&scheme=1)

[Click here to access/download](https://www2.cloud.editorialmanager.com/pone/download.aspx?id=37099183&guid=ab1876cf-92ff-4776-8e08-69dbc4cd4dd2&scheme=1)

[Supporting Information](https://www2.cloud.editorialmanager.com/pone/download.aspx?id=37099183&guid=ab1876cf-92ff-4776-8e08-69dbc4cd4dd2&scheme=1)

[S2 Fig_Example Australian CTC cost template .docx](https://www2.cloud.editorialmanager.com/pone/download.aspx?id=37099183&guid=ab1876cf-92ff-4776-8e08-69dbc4cd4dd2&scheme=1)

1

1 **Australian Communities That Care (CTC) intervention: Benefit-cost analysis of a**
2 **community-based youth alcohol prevention initiative**
3 **Julie Abimanyi-Ochom^1&#*^, Sithara Wanni Arachchige Dona^1&*^, Shalika Bohingamu Mudiyanselage^1^,**
4 **Kanika Mehta^2^, Margaret Kuklinski^3^, Bosco Rowland^4&5^, John W Toumbourou^4^ and Rob Carter^1¶#^**

5

6 ^1^ Deakin Health Economics, School of Health and Social Development, Institute for Health Transformation,
7 Faculty of Health, Deakin University, Geelong, Victoria, Australia
8 ^2^ School of Medicine, IMPACT – The Institute for Mental and Physical Health and Clinical Translation, Deakin
9 University, Geelong, Victoria, Australia
10 ^3^ Social Development Research Group, School of Social Work, University of Washington, Seattle, Washington,
11 USA
12 ^4^ School of Psychology, Centre for Social and Early Emotional Development, Faculty of Health, Deakin
13 University, Geelong, Victoria, Australia
14 ^5^ Eastern Health Clinical School & Monash Addiction Research Centre, Monash University, Victoria, Australia 15
16 ^¶^#Shared Joint senior authorship

Formatted: Superscript

17 ^&^*Shared Joint first authorship 18

Formatted: Superscript

19

20

21

22

23

24

25

26

27

28

29

30

31

32 **^*^Corresponding Author**

Formatted: Superscript

33 Julie Abimanyi-Ochom
34 Deakin Health Economics, School of Health and Social Development, Institute for Health Transformation, Faculty
35 of Health, Deakin University, Geelong, Victoria, 3220, Australia
36 Email: [j.abimanyiochom@deakin.edu.au](mailto:j.abimanyiochom@deakin.edu.au)

37

38

39

40

41	**Abstract**

42 **Background:** Internationally, ~~A~~adolescent alcohol consumption has been a major community
43 concern for decades. Globally, there is a growing array of interventions aimed at preventing
44 youth alcohol-related problems. Notably, the Communities that Care (CTC) process in the USA
45 has proven to be a cost-effective intervention, leading to a reduction in adolescent alcohol-
46 related problems. In Australia, the CTC trial has shown positive outcomes such as reduced
47 adolescent alcohol and drug use, antisocial behaviors, and the availability of alcohol in the
48 community. To encourage wider adoption, it is essential to ascertain the cost-effectiveness of
49 the Australian CTC trial and its potential impact on addressing youth alcohol problems.
50 **Method:** We conducted a societal perspective benefit-cost analysis focused on reducing
51 adolescent (aged 10-14) alcohol use in the initial four CTC communities in Australia, spanning
52 2001 to 2015. To ensure accuracy, estimated benefits were adjusted for joint effects to prevent
53 the double counting of the benefits over time.
54 **Results:** The Australian CTC trial, as an adolescent alcohol prevention strategy, demonstrates
55 robust economic credentials, delivering a return of AUD 2.6 for each dollar invested. The cost
56 savings resulting from the reduction in alcohol consumption were estimated at AUD 123 per
57 youth per 15 years and AUD 8 per person per year. The average cost of the CTC trial amounted
58 to AUD 48 per youth over 15 years and AUD 3 per youth per year. The largest contribution to
59 primary benefits were reductions in crime and violence with 93% of the total benefits.
60 **Conclusions:** This study makes a valuable contribution to the international economic evidence
61 related to youth alcohol prevention initiatives. The results affirm the strong cost-effectiveness
62 potential of the initial CTC trial implementation in Australia. Moreover, conducting further
63 cost-benefit modelling for additional outcomes and beyond the intervention’s timeframe is
64 likely to enhance the economic viability of the CTC trial.

Formatted: Font: 18 pt

Formatted: Left

65 ***Keywords:*** cost-benefit analysis, community-based intervention, Australian Communities That
66 Care, youth alcohol prevention, CTC program Australia.

### 67 Introduction

68 Globally, excessive alcohol consumption is a leading risk factor for death and disability ^1^.
69 Alcohol consumption often begins during the early teenage years, and risky levels of
70 consumption during the teen years are associated with harmful consumption in the adult years
71 ^2^. Australia has the highest rates of adolescent alcohol use in the world ^3^. If alcohol related
72 health and community issues are to be avoided, cost-effective programs and policies need to
73 be systemically implemented ^4^. Australia implemented the Communities That Care (CTC)
74 prevention framework with the aim of reducing and preventing underage alcohol consumption
75 ^4^. The CTC framework can also be described as a preventive intervention.
76 The CTC framework was originally developed in the United States (US) and was later
77 implemented as a community intervention in Australia in 2002 (trial-related prior-
78 implementation activities happened prior to 2002) ^5^, which aimed at reducing adolescent
79 alcohol and drug use by identifying and addressing local risk and protective factors ^6^.
80 CTC is designed to build prevention capacity and sustainability across large municipal
81 communities and is implemented in 5 phases ^6-8^. Communities begin by forming a municipal
82 coalition of stakeholders (Phase 1 & 2). They then collect local data and develop a profile of
83 their community (Phase 3) to identify elevated risk factors that cause adolescent alcohol use
84 (e.g., parent and community supply of alcohol, low perceived risk, poor family management).
85 Communities then use this profile to inform the selection of evidence-based strategies that
86 target elevated risk factors (e.g., programs to reduce underage alcohol supply, parent
87 education), while also increasing protective factors such as community rewards and
88 opportunities (Phase 4). Communities then implement and evaluate evidence-based strategies
89 (Phase 5), and monitor their progress by repeating the survey again, and comparing to their
90 Phase 3 results. The CTC trial was conducted in four Australian community coalitions (Local
91 Government Areas (LGA) across Australia (in Victoria and Western Australia) from 2002 to
92 2015. The evaluation compared 104 non-CTC LGAs ^6^. The CTC trial in Australia evaluated
93 data collected over a 15-year period.
94 The CTC trial action plans focused on reducing family risk factors and favorable attitudes to
95 alcohol and drugs in youth using school-based and parent/family drug education ^9,10^. ~~Appendix~~
96 Supporting information S1 outlines the number of CTC cycles and the duration of the CTC
97 trial in the first four community coalitions. Community coalition 1 completed two cycles
98 compared to the other three communities, which completed only one cycle each.
99 The CTC trial in US has demonstrated reductions in adolescent alcohol and drug use, and
100 related antisocial behaviors, less favorable parent attitudes toward alcohol and drug use, and
101 reduced community availability of alcohol ^11,12^. Given these novel findings, this study aims to
102 develop a benefit-cost analysis model to explore the cost and benefits from a societal
103 perspective (i.e., all costs and outcomes to whomever they accrue which includes health sector
104 (public/private) or governments or individuals/patients/families, or taxpayer or third party
105 funders ^13^) using data from a 15-year evaluation of the Australian CTC intervention in 4 trial
106 communities. Benefit-cost analysis will inform CTC trial effectiveness and guide policymakers
107 and stakeholders interested in achieving positive youth outcomes in a cost-effective way.
108 A benefit-cost analysis places a monetary value on significant intervention-related outcomes,
109 based on benefits expected to accrue over the reference period for the participants. The present
110 economic analysis of the Australian CTC trial seeks to determine whether the benefits
111 associated with the observed effects of the CTC trial outweigh the costs of implementation.

Formatted: Heading 2, Left

Formatted: Heading 2, Left

Formatted: Heading 1, Left, Line spacing: single

112 The benefit-cost analysis results provide economic evidence related to the CTC trial and return
113 on investment of public dollars, even under very conservative cost and benefit assumptions.

### 114 Material and methods

115 The benefit-cost model estimated the cost of the intervention and the benefits in the youth
116 population based on the reduction in alcohol use due to the CTC trial. Firstly, the reduction in
117 alcohol use measured in the trial was used to estimate the reduction in the alcohol consuming
118 population due to the intervention for the intervention population. Secondly, we estimated the
119 population that have risky alcohol consumption within the alcohol consuming intervention
120 population. Lastly, avoided consequences in health-related events (alcohol-related hospital
121 admissions, emergency department visits, ambulance attendances, alcohol treatment
122 programs), and in non-health events (deaths, and crime) due to risky alcohol consumption were
123 estimated and costed to give the benefit from the CTC intervention.

124	**Study design**

125

126	An intervention-based benefit-cost model was conducted from a societal perspective.

127	**~~Model data~~**

128	**Model period**

129 The periods for the benefit-cost model were derived using the Australian Bureau of Statistics
130 (ABS) data periods, which provides five-year population data estimates. ^14-16^. This provided
131 three model periods covering 15 years of the trial: Period 1; 2001-2005, Period 2; 2006-2010
132 and Period 3; 2011-2015.

#### 133 Model Population

134 The ABS population numbers were aggregated by CTC trial LGAs, age, sex, and year ^14-16^.
135 The ABS five-year population numbers were combined with survey estimates of the proportion
136 of alcohol consumption 17 for each age group to estimate the population who do consume
137 alcohol (Figure 1).

138

139	[Insert Figure 1: Model population 2001-2015]

140

141

142

143	**Model inputs**

144 Through interpolation of results of Toumbourou and colleagues study, the Australian CTC trial
145 demonstrated a larger reduction in adolescents using alcohol of 28.3% compared to control
146 communities with a 12.2% reduction over 15 years ^6^. The difference in reduction in alcohol use
147 between the two groups was 16.1 percentage points, or an average absolute reduction in use of
148 1.01% per year (i.e., 1.01= 16.1/15, 5.05% per five years) ^6^. We considered a single occasion
149 of alcohol risk ^18^, which is more applicable to the model population. Details of the model inputs
150 are shown in Table 1.

151

152 [insert table 1: Model inputs]
153 Table 1: Benefit-cost model inputs

| **Model input** | **Model Value** | **Reference and notes** |
| --- | --- | --- |
| Average absolute reduction in alcohol use due to CTC trial | 1.01% per year | Toumbourou et al., 2019 ^6^ |
| Percentage of adolescents that use alcohol (10-17 years  old) | 61.8% | Kelly et al., 2015 ^17^ |
| Percentage of adolescents within the intervention community exposed to the CTC trial | 100% | Kuklinski et al., 2012 ^19^ |
| **Alcohol risk**  Percentage of youth using alcohol at levels that risk harm: 2006-2010 | 18-19 years old  Males: 18.5%,  Females: 15.9% | 2007 AIHW estimated alcohol- related harm from a single occasion (more than 4 drinks) by age and sex  (17.2% gender aggregated rate) ^20^ |
| **Alcohol risk**  Percentage of youth using alcohol at levels that risk harm: 2011-2015 | 18-19 years old  Males: 13.1%,  Females: 15.8%  20-24 years old  Males: 13.1%,  Females: 15.8% | 2013 AIHW alcohol-related harm from a single occasion (more than 4 drinks) by age and sex (gender aggregated rate: 18-19 years old - 8.3%, 20-24 years old – 14.3%) ^21^ |
| Percentage of youth alcohol-related emergency department visits and cost per visit | 2001, 2006, 2011:  15.1%, 24.4%, 28%  $290 (2005) | 15-24 age rate used, cost ^22,23^ |

| Percentage of youth alcohol-related hospital admissions and cost per admission | 2001, 2006, 2011:  29.3%, 39.9%,  47.6%  $22,375 (2005) | 15-24 age rate used, cost ^22,23^ |
| --- | --- | --- |
| Percentage of youth alcohol related ambulance attendance and cost per attendance | 2001, 2006, 2011:  17.9%,17.9%,56.2%  $689 (2012) | 15-24 age rate used, cost ^23,24^ |
| Percentage of youth alcohol related alcohol treatment programs and cost per program, | 2001, 2006, 2011:  45.4%, 60.5%,  50.3%  $7,285 (2011) | 15-24 age rate used, cost ^23,25^ |
| Percentage of youth alcohol-related deaths and cost per life lost | 2001, 2006, 2011:  0.67%, 0.67%,  0.52%  $1,365,563 (2010) | 15-24 age rate used, cost ^23,26^ |
| Number of overall crimes (age 10-19) | 109,844 (age 10-19) | Offenders (2008-09) ^27^ |
| Percentage of offenders involved in alcohol-related incidents | 1.4% (age 10-14),  18.8 (age 15-19) and  25.4% (age 20-24) | 2007 ^28^ |
| Alcohol attributable fraction of crime | 15% | in 2008, 2010 & 2015 ^29^ |

154 Note: AIHW- Australian Institute of Health and Welfare

155

156	**Model outputs**

Formatted: English (United States)

157

158 Model outputs are presented as follows: (1) The Australian CTC trial cost, (2) short-term cost
159 savings (i.e., benefits) from avoided events to health, and criminal system due to CTC trial,
160 and (3) Benefit-Cost ratio (BCR) as primary outcome of CTC trial.

161	**Data Analysis**

Formatted: Heading 2, Left

162 The cost and benefits estimation methods are presented in Table 2. First, the cost of the CTC
163 trial was calculated for each of the four CTC trial communities using CTC trial records
164 provided by the trial implementation team using a template designed by the health economics
165 investigators (See S2 Example anonymised Australian CTC community cost template). CTC
166 costs were attributed to organizing the community coalitions, intervention programs, student
167 surveys, rental space, salary, CTC training for each of the 5 phases and other costs such as
168 transport, catering resources, and report development.

Activity costs (categories) were

Formatted: Highlight

169

Formatted: Highlight

estimated using an approach ^26^ of multiplying the quantity of each resource by its unit price to

170 generate a resource cost. The costs of all resources used in an activity were then summed ~~up~~ to

Formatted: Highlight

171 give the total cost of the activity. The activity costs were summed up to give the total cost per
172 community for the intervention period. Only the first cycle was taken into account when there
173 were multiple cycles during the intervention period (see S2 Example anonymised Australian
174 CTC community cost template).
175 Second, societal benefits from averted events resulting from the reduced alcohol consumption
176 due to the CTC trial were modelled and calculated for the 15-year intervention period ^30^. The
177 model assumption was that the reduction in alcohol consumption due to the CTC trial 6 led to
178 the reduction in alcohol-related adverse events and associated costs during the intervention
179 period.
180 The Australian low-risk drinking guidelines recommend that adolescents should not consume
181 alcohol before the age of 18 ^31^. Thus, the model assumed that for the 10 to 17-year-old
182 population, any alcohol consumed is harmful. For the 18 to 19-year-olds and 20 to 24-year-
183 olds alcohol was assumed harmful when consumed above the NHMRC (National Health and
184 Medical Research Council) recommended levels, more than four standard drinks in a session.
185 Avoided adverse events for alcohol users were categorised as health and non-health related. As
186 shown in Table 2, the health-related avoided events include: 1) alcohol-related hospital
187 admissions, 2) emergency department (ED) visits, 3) ambulance attendances, 4) alcohol
188 treatment programs. The non-health events include: (1) deaths, and 2) crime.
189 The benefits were adjusted for joint effects using a base model of 50%, where the total benefits
190 were halved, based on previous literature^32^adjusted for possible double counting due to
191 correlations among benefits from different outcomes ^33^. The joint effect was considered to
192 correct for shared risk factors that can contribute to the same outcomes. All nominal costs and
193 benefits estimates were converted to 2020 Australian dollars (AUD) using the health price
194 index ^34,35^. The model was generated using Microsoft Excel. The impact of intangible costs
195 and benefits such as stress and quality of life were not included in the model. The total, average,
196 weighted average intervention cost, total and average benefits, and average dollars saved per
197 youth were calculated. The benefit-cost ratio was calculated as the ratio of the average dollar
198 saved per youth and average cost per youth.

199

200

201	Table 2: Cost and benefits estimation methods

| **Costs and benefits** Measures Estimations and assumptions Data source Cost and benefits estimation Cost and benefits estimations  method | | | | | |
| --- | --- | --- | --- | --- | --- |
| **Intervention cost** | Total intervention | Expenditure *Categories* | CTC implementation | CTC trial cost for each | Total CTC trial cost for each |
|  | implementation cost | 1. Administration cost | team | expenditure category per ABS | expenditure category. |
|  |  | (organizing the community |  | youth aged 10-14 years who lived |  |
|  |  | coalitions intervention |  | in the study communities in 2000- | The average cost per |
|  |  | programs, student surveys) |  | 2015 | community weighted by |
|  |  | 2. Rental space cost |  |  | community size (weighted |
|  |  | 3. Staff salary |  |  | average), which reduces the |
|  |  | 4. CTC training for each of the |  |  | impact of population size on |
|  |  | five phases |  |  | average cost for small |
|  |  | 5. Transport |  |  | population communities. |
|  |  | 6. Catering resources |  |  |  |
|  |  | 7. Report development |  |  |  |
|  |  |  |  |  |  |
| **Benefits of the** |  |  |  |  |  |
| **intervention** |  |  |  |  |  |
| **Avoided health** |  |  |  |  |  |
| **system events** |  |  |  |  |  |
| **alcohol-related**  **Emergency** | 1. Rate of avoided youth  alcohol cases, 5.05% for |  | Toumbourou et al.,  2019 ^6^ | The total population in the CTC  communities x 5.05% | Total number of avoided  youth alcohol cases in the |
| **Department (ED)**  **presentations** | five years  2. Percentage of alcohol- | The proportion of alcohol- | Turning point ^23^ | (Total number of avoided youth | CTC communities |
|  | attributed injuries at EDs, | attributed injuries presented at |  | cases in the CTC communities | Total number of avoided |
|  | 15.1%, 24.4% & 28.0% | EDs in 2001, 2006 & 2011 |  | (5.05%/5-years) x the proportion | alcohol-attributed ED |
|  |  |  |  | of alcohol-attributed injuries | presentations |
|  |  |  |  | presented at EDs per 10,000 |  |
|  |  |  |  | population) |  |
|  | 2. Cost per ED presentation, | Average ED treatment costs for | Turning point ^23^ | Total number of avoided alcohol- | The total cost saved from |
|  | $ 254 | all states and territories, |  | attributed ED presentations x $254 | avoided alcohol-attributed ED |
|  |  | considering both acute and |  |  | presentations |

non-acute ED expenses

**Costs and benefits**

Measures

Estimations and assumptions

Data source

Cost and benefits estimation method

Cost and benefits estimations

**alcohol-related hospital admissions**

1. Rate of avoided youth alcohol cases, 5.05% for five years

Toumbourou et al., 2019 ^6^

~~Kelly et al., 2015~~ ^17^

The total population in the CTC communities x 5.05%

Total number of avoided youth cases in the CTC communities

2. Percentage of alcohol- attributed hospital admissions, 29.3%, 39.9%

& 47.6%

The proportion of alcohol- attributed hospital admissions Eds in 2001, 2006 & 2011

Turning point ^23^	(Total number of avoided youth

cases in the CTC communities x the proportion of alcohol- attributed hospital admissions per 10,000 population)

Total number of avoided alcohol-attributed hospital admissions

2. Cost per hospital admission, $ 22,357

Average hospital admission treatment cost

Laslett et al., 2010 ^22^	Total number of avoided alcohol-

attributed hospital admissions x

$22,357

The total cost saved from avoided alcohol-attributed hospital admissions

**alcohol-related ambulance attendances**

1. Rate of avoided youth alcohol cases, 5.05% for five years

Toumbourou et al., 2019 ^6^

~~Kelly et al., 2015~~ ^17^

The total population in the CTC communities x 5.05%

Total number of avoided youth cases in the CTC communities

2. Percentage of alcohol-

attributed ambulance attendance, 17.9%, 17.9% &

56.2%

The proportion of alcohol-

attributed ambulance attendance in 2001, 2006 &

2011

Turning point ^23^	(Total number of avoided youth

cases in the CTC communities x the proportion of alcohol- attributed ambulance attendance per 10,000 population)

Total number of avoided alcohol-attributed ambulance attendance

2. Cost per ambulance attendance, $ 689

Average ambulance attendance cost

Watts et al., 2013 ^24^	Total number of avoided alcohol-

attributed hospital admissions x

$689

The total cost saved from avoided alcohol-attributed ambulance attendance

**alcohol treatment programs**

1. Rate of avoided youth alcohol cases, 5.05% for five years

Toumbourou et al., 2019 ^6^

The total population in the CTC communities x 5.05%

Total number of avoided youth cases in the CTC communities

2. Attendance percentage for alcohol-attributed treatment programs 45.4%, 60.5% &

50.3%

Participation proportion for alcohol-attributed treatment programs in 2001, 2006 &

2011

Turning point ^23^	(Total number of avoided youth

cases in the CTC communities x the participation proportion for alcohol-attributed treatment programs per 10,000 population)

Total number of avoided participations in alcohol- attributed treatment programs

**Costs and benefits**

Measures

Estimations and assumptions

Data source

Cost and benefits estimation method

Cost and benefits estimations

|  | 1. Treatment cost per case, | The average treatment cost per | Smith et al., 2014 ^25^ | Total number of avoided | The total cost saved from |
| --- | --- | --- | --- | --- | --- |
|  | $7285 | case: residential drug |  | participations in alcohol-attributed | avoided participants in |
|  |  | treatment, non-residential |  | treatment programs x $7285 | alcohol-attributed treatment |
|  |  | treatment and |  |  | programs. |
|  |  | pharmacotherapeutic |  |  |  |
|  |  | treatments |  |  |  |
| **Avoided non-health** |  |  |  |  |  |
| **system events** |  |  |  |  |  |
| **overall crimes** | 1. Rate of avoided alcohol  consumption, 5.05% for five |  | Toumbourou et al.,  2019 ^6^ | The total population in the CTC  communities x 5.05% | Total number of avoided  youth cases in the CTC |
|  | years  2. % of offenders by age |  | Palk et al., 2007 ^28^ | Total number of avoided youth | communities  Total number of avoided |
|  | involved in alcohol- |  |  | cases in the CTC communities x | crimes |
|  | attributed incidents  3. Alcohol attributable crime | Alcohol attributable crime cost | Collins et al., 2008 ^29^ | % of offenders by age  Alcohol attributable crime cost in | The total cost saved from |
|  | cost per person, $28,603.66 | in 2004-05 |  | 2004-05 x total number of avoided | avoided crimes |
|  |  |  |  | crimes |  |
| **alcohol-related deaths** | 1. Rate of avoided alcohol consumption, 5.05% for five |  | Toumbourou et al., 2019 ^6^ | The total population in the CTC communities x 5.05% | Total number of avoided youth cases in the CTC |
|  | years  2. Percentage of alcohol- | The proportion of alcohol- | Turning point ^23^ | (Total number of avoided youth | communities |
|  | attributed deaths, 0.67%, | attributed deaths in 2001, 2006 |  | cases in the CTC communities x | Total number of avoided |
|  | 0.67% & 0.52% | & 2011 |  | the proportion of alcohol- | alcohol-attributed deaths |
|  |  |  |  | attributed deaths per 10,000 |  |
|  |  |  |  | population) |  |
|  | 2. Cost per death, | Average cost per incident | Manning et al., 2013 | Total number of avoided alcohol- | The total cost saved from |

202

203

204

$1,367,230

(reduced workforce and	^26^

reduced household labor from premature mortality)

attributed deaths x $1,367,230

avoided alcohol-attributed deaths

14

Formatted: Font: 18 pt

Formatted: Left

Formatted: Normal, Left

Formatted: Heading 2, Left

#### 205 Sensitivity analysis

206 A sensitivity analysis was undertaken to deal with uncertainty in average absolute reduction
207 due to the CTC trial ~~and~~ to ensure the reliability of the BCR results and conclusions.
208 Bootstrapping was used for sensitivity analysis for the model variables. The ‘average absolute
209 reduction’ of 1.01% per annum was modelled to account for fading intervention effects with ±
210 5 and 10% variation. The variation of the proportion of youths exposed to the intervention was
211 ± 5 and 10% of the base model. Similarly, the variation of other benefits variables was also
212 applied through the sensitivity analysis.

213	**Results**

214

215 The total youth population aged 10-14 in the four community coalitions assumed to be
216 impacted during the intervention period from 2001 to 2015 was 48,210.

217

218	**CTC costs**

219	The total cost of implementing the Australia CTC trial for the four pioneer community

220	coalitions over 15 years was estimated at AUD 2.3 million (Table 3). The majority of total

221	intervention costs were attributed to staff salary allocations (42%). Considering only the first

222	cycle for all included community coalitions, community coalition 2 had the highest intervention

223	cost estimated at AUD 0.70 million, and community coalition 4 had the lowest intervention

224	cost, estimated at AUD 0.42 million. The CTC trial cost per youth was AUD 48 for the entire

225	intervention period and AUD 3 per year per youth.

226

227	[Insert Table 3]

Deakin - External Collaboration

15

228

229

###### 230 Table 3: CTC trial costs by cost category (AUD2020)

231

|  | **Duration of CTC (Years)** | **Cost categories AUD2020** | | | | | | | **Population** | **Total CTC trial cost** | **Total cost per youth** | **Total cost per year per youth** |
| --- | --- | --- | --- | --- | --- | --- | --- | --- | --- | --- | --- | --- |
|  |  | **Community mobilization cost** | **CTC trial program cost** | **Student survey cost** | **Rental space cost** | **Salary allocation costs** | **CTC**  **training cost** | **Other costs**  **including transport** |  |  |  |  |
| Community coalition 1 | 6 | 173,892 | 17,602 | 49,724 | 7,661 | 201,755 | 1574,33 | - | 28,003 | 608,067 | **8.74** | **0.58** |
| Community coalition 2 | 13 | 121,432 | 15,626 | 88,249 | 8,616 | 280,826 | 186,802 | - | 12,199 | **704,799** | 12.61 | 0.79 |
| Community coalition 3 | 10 | 89,387 | 15,626 | 65,058 | 5,607 | 270,593 | 127,265 | 5,949 | 4,409 | 579,484 | **14.62** | 0.79 |
| Community coalition 4 | 7 | 46,066 | 36,096 | 27,153 | 7,421 | 220,770 | 83,680 | - | 3,599 | **421,187** | 12.02 | **1.12** |
| **Total** |  | 430,777 | 84,950 | 230,184 | 29,305 | 973,944 | 555,180 | 5,949 | 48,210 | 2,313,537 | 48 | 3 |
| **Proportion of total**  **CTC trial cost** |  | 19% | 4% | 10% | 1% | 42% | 24% | 0.3% | - | - | - | - |

232 Note: Costs are reported only for the first cycle when there are two cycles (community coalition 1). Bold figures represent the highest and lowest costs. The intervention
233 costs were provided by the CTC trial team (based on actual expenses during the trial period, (considering the unit costs of each cost category for each community coalition)).
234 The intervention costs accounted for all the costs relevant to delivering the intervention including on-costs.

Deakin - External Collaboration

16

Formatted: Heading 2, Left

235 Regarding the CTC trial cost per youth, community coalition 1 had the lowest average
236 intervention cost, with AUD 8.74 per youth, while community coalition 3 had the highest cost,
237 at AUD 14.62 per youth. In terms of the cost per youth per year for the CTC trial, community
238 coalition 1 had the lowest average intervention cost, at AUD 0.58 per youth per year, while
239 community coalition 4 had the highest annual cost, at AUD 1.25 per youth.

#### 240 CTC overall benefits

241 The avoided cost from alcohol-related events resulting from the CTC trial was estimated at
242 AUD 5.9 million (as detailed in Table 4) over the 15-year period from 2001-2015. Notably, the
243 highest avoided cost was attributed to alcohol-related crimes at AUD 5.5 million (93%), while
244 the lowest was associated with ED visits, totaling AUD 1,360 (< 1%). On average, the benefit
245 (avoided cost), per youth was AUD 123 for the entire intervention period and AUD 8 per year.
246 [Insert Table 4]

Deakin - External Collaboration

17

###### 247 Table 4: CTC trial benefits (avoided costs) (AUD2020) (adjusted for joint effects)

| **CTC trial benefits (avoided costs due to alcohol consumption reduction)** | **Total benefit (AUD)** | **Benefit percentage of total benefits** | **Total benefit per youth**  **for the intervention period (AUD)** | **Benefit per**  **youth per year (AUD)** |
| --- | --- | --- | --- | --- |
| **Health-related benefits** | 281,470 | 4.8% | 5.8 | <1 |
| Alcohol-related hospital admissions | 204,747 | 3.5% | 4.2 | <1 |
| Alcohol-related ED visits | 1,360 | <1% | <1 | <1 |
| Alcohol-related ambulance attendance | 3,905 | <1% | <1 | <1 |
| Alcohol-related treatment programs | 71,458 | 1.2% | 1.5 | <1 |
| **Non-health related benefits** | 5,638,549 | 95.3% | 117 | 7.8 |
| Alcohol-related deaths (Mortality) | 157,767 | 2.7% | 3.3 | <1 |
| Alcohol-related all crimes | 5,480,782 | 92.6% | 113.7 | 7.6 |
| **Total benefits** | 5,920,018 | 100% | 123 | 8 |

248

249

Deakin - External Collaboration

Formatted: Font: 18 pt

Formatted: Left, Line spacing: single

Formatted: Heading 2, Left, Line spacing: single

250 Ninety-five percent of benefits were from non-health related avoided costs, while the other 5%
251 were from health-related avoided costs. According to the estimated total cost and total benefits,
252 the benefit cost ratio is 2.6, which translates to a return of AUD 2.6 for each dollar invested.

#### 253 Sensitivity analysis

254 The sensitivity analysis found the model outputs to be robust. The BCR marginally differed for
255 each variable with ± 5 and ±10 variations. The lowest BCR was estimated at 2.34 when the
256 average absolute reduction was assumed to -10% of 1.01, while the highest BCR was at 2.86
257 when the average absolute reduction was assumed to +10% of 1.01.

258	**Discussion**

259 This study reports the benefits and cost of the inaugural Australian CTC trial undertaken with
260 4 CTC trial communities, revealing that each dollar spent on the CTC trial per youth per year
261 resulted in an estimated savings of AUD 2.6, primarily attributed to avoided alcohol-related
262 crime events. These findings offer valuable insights into quantifying and monetizing the
263 benefits of community-wide adolescent alcohol prevention initiatives within the Australian
264 context. They underscore the potential of the CTC prevention framework to improve adolescent
265 health and behavioral outcomes in Australia.
266 Intervention costs are lower in Australia, compared to the US ^19^. The Australian CTC trial cost
267 was AUD 3 per youth per year compared to AUD 199 per youth per year for the US CTC trial
268 (2004 USD 513 per youth for 5 years/ 102 per youth per year). The main reason for a lower
269 cost in the Australia CTC was~~could be~~ that community coalition service delivery budgets were
270 redeployed in Australia compared to the US where new budgets were introduced for the CTC
271 trials. In Australia there was also a greater use of community alcohol supply reduction
272 interventions (e.g., underage test purchasing, retailer warning letters and media releases) which
273 were less costly than the family and school interventions used in the US.
274 It is not entirely comparable to contrast the US return on investment with the finding of return
275 of investment for this Australian study. T~~he return on investment is lower in Australia,~~
276 ~~compared to the US~~. The return for the US CTC trial was AUD 10.3 (2004 USD 5.3) for each
277 dollar spent compared to a return of AUD 2.6 for each dollar spent in the Australian CTC trial
278 ^19^. However, the US analysis included both the long (lifetime) and short term (limiting to
279 intervention period) harms prevented from tobacco use, delinquency, and school dropout ^36^.
280 The Australian CTC trial demonstrated significant prevention effects on adolescent lifetime
281 alcohol, tobacco, and cannabis use and past year antisocial behavior [10]. However, the current
282 BCR analysis was conservative as it only included impacts associated with reduced alcohol
283 consumption over the intervention period of 15 years. Importantly, this analysis demonstrates
284 that preventive interventions can incur significant savings related to youth alcohol use over 15
285 years. Other Australian studies examining the benefit-cost of reducing alcohol consumption
286 from a societal perspective have identified larger life-time cost savings as alcohol causes high
287 costs to the society ^29,36^. However, these studies have examined adult populations and included
288 both long- and short-term economic impacts at the population level ^29^. Given that our study
289 only examined benefits during the intervention period using community level data, modelling
290 lifetime benefits may identify greater benefit-cost savings for Australian communities, as
291 evidenced from the US CTC evaluation ^19^.

292

293 The major component of costs for implementing CTC in this trial was related to staff and
294 training. This training focused on developing stakeholder knowledge of prevention science and
295 teaching them how to collect and critically interpret appropriate prevention data. The data
296 helped them strategically and systematically prioritize behavioral outcomes and recognize and
297 implement tested effective programs ^37^. As the CTC trial is also designed to build community
298 capacity, the costs needed to implement the CTC trial will likely diminish over time. In the
299 subsequent CTC cycles, communities will require less training to progress through the CTC
300 phases or will be able to facilitate their own training with minimal support. As an example, in
301 the second cycle for the community coalition community with two cycles, the cost was reduced
302 by AUD 65,736.

303

304 Most community coalition alcohol prevention programs were funded by redeploying or
305 reallocation of existing resources. Therefore, the opportunity costs of the Australian CTC trial
306 implementation were lower compared to the few communities that implemented utterly new
307 programs. This was particularly important for the feasibility of the CTC trial implementation
308 in small communities with smaller populations and fewer resources (the sample ranged from
309 3,599 to 28,003 in the four community coalition communities). This suggests that return on
310 investment for implementing the CTC framework can be partly linked with assisting
311 community investment to reorient health and evidence-based prevention services to meet
312 community needs more efficiently, rather than implementing and developing entirely new
313 services. It suggests that mobilizing communities to work within a prevention framework to
314 implement evidence-based programs or reorient services can be cost-beneficial. The
315 implementation of CTC in Australia is mainly through the re-allocation of existing resources,
316 which should help with the sustainability of CTC over time. A call for communities to reorient
317 health services has been advocated since the launch of the Ottawa Charter for Health
318 Promotion, in 1986 ^38^.
319 Other community prevention interventions, which used experimental designs, have also
320 demonstrated efficacy in preventing adolescent alcohol use ^39^. Like CTC, these interventions
321 depended on mobilizing communities and long-term prevention investment. Thus, the findings
322 of our study suggest that further examination of the economic benefits of CTC using an
323 experimental design would be a worthy investment. If possible, it would be essential to

Formatted: Heading 2, Left

324 compare the average absolute reduction of the BCR for different community approaches and
325 identify whether a particular approach is more effective and efficient. Based on the model
326 results, the CTC trial had a positive impact on crimes due to reduction in alcohol consumption
327 among youths and therefore continuation of these kinds of interventions could significantly
328 reduce crime.

329

330 A strength of the study is its naturalistic evaluation context and, therefore, its high ecological
331 validity. It used data collected over a 15-year period to evaluate trends that are influenced by
332 real life factors and community demands. Moreover, the sensitivity analysis demonstrated the
333 results are robust with marginal changes in BCR, with the Australian CTC trial still
334 worthwhile (cost beneficial overall) after adjustment of the different rates used in the benefits
335 model.

336

#### 337 Limitations and implications

338 The Australian CTC trial study was conducted as a pragmatic community coalition trial, ~~and~~
339 with 4 trial communities, and these communities were not randomized. Data sources used did
340 not account for differences in rates of alcohol consumption and alcohol-related events by state
341 (e.g. Victoria and Western Australia) ^40^. Thus, the model may have underestimated CTC trial
342 benefits for Western Australian communities, since alcohol consumption is generally higher in
343 these communities ^41^.

344

345 Alcohol attributable event rates by age and year of occurrence were used to estimate benefits
346 from reduced ED, hospital admission, ambulance attendance, treatment programs, and death.
347 It is possible that using the same rate by gender may have overestimated benefits for females
348 and underestimated benefits for males. Furthermore, rates on ambulance attendance used
349 2006/07 rates for missing 2001 values, and rates on death used 2001 rates for missing 2006
350 rates, which may have underestimated and overestimated benefits respectively.
351 Alcohol consumption rates vary by gender and age, but this was not accounted for based on
352 data available on the CTC trial average absolute reductions in the reference sources^6^. Using an
353 average ~~average~~ absolute reduction may have overestimated or underestimated the average
354 absolute reduction for females or males, respectively. The annualized rate of the average
355 absolute reduction over the intervention period does not account for the fading effect of the
356 intervention. However, sensitivity analysis explored uncertainty around the average absolute
357 reduction, and the BCR was still greater than 1, hence even with a possible fading intervention
358 effect, the CTC trial was still judged to be beneficial.

359

360 Similarly, the alcohol attribution fraction of crime was consistent across the period, which
361 probably led to underestimating the benefits. The measured police-reported crime prevention
362 benefit in the relevant Australian CTC evaluation 42 is consistent with that attributed in the
363 current analysis. The national rates of alcohol-related harm may not be specific to the CTC
364 communities. Rates associated with alcohol-related events may include harm due to youth
365 alcohol use and harms to youths due to other alcohol users. The model adjusted for joint effects,
366 by multiplying benefits by a factor of 0.5 using ^32^. However, there is limited evidence on
367 alternative methods to adjust for joint effects. The model inputs- rates for our study were based
368 on the literature (except for alcohol reduction rates). Various benefits typically included in
369 similar analyses, especially for long term or lifetime impacts, were not included in this analysis.
370 This included factors such as effects on school dropouts, employment and intangible benefits.
371 Therefore, future research on the Australian CTC should aim to estimate long term or lifetime
372 benefits by incorporating these benefits.

Formatted: Font: 18 pt

Formatted: Left

Formatted: Font: 18 pt

Formatted: Heading 1

Formatted: Heading 2

373	**Conclusion**

374

375 The benefit-cost ratio of the Australian CTC trial was found to be 2.6, which demonstrates that
376 benefits (avoided alcohol-related costs) outweighed the Australian CTC trial cost across 15
377 years. The study suggests that the CTC trial implementation is worthwhile. Findings also
378 suggest that CTC offers a cost-beneficial evidence-based approach for use by coalition
379 communities, governments, and large funding bodies to bring about population change in
380 alcohol-related health and social outcomes. This paper extends the range of cost-benefit
381 interventions available in the international context to prevent adolescent health and social
382 problems. Future research should incorporate the economic benefits of prevented youth
383 tobacco and cannabis use and antisocial ~~behaviour~~behavior demonstrated in the Australian
384 CTC Trial 6 and incorporate more robust experimental designs, average absolute reductions
385 that accounts for age, gender, length of intervention period, and long-term benefits.

386

#### 387 Conflict of Interest: None

388

389 ~~Funding acknowledgement: Bosco Rowland, John W Toumbourou and Rob Carter received~~
390 ~~funding from the National Health and Medical Research Council (APP1087781) and~~
391 ~~Australian Research Council (LP100200755).~~

392

| 393 | **~~Appendix~~Supporting information** |
| --- | --- |
| 394 |  |
| 395 | S1: Duration of the Australian CTC implementation |
| 396 | S2: Example anonymised Australian CTC community cost template |

397

Formatted: Heading 1, Space Before: 0 pt

398

399	**References**

400

401 1.	Griswold MG, Fullman N, Hawley C, et al. Alcohol use and burden for 195 countries and territories,
402 1990–2016: a systematic analysis for the Global Burden of Disease Study 2016. The Lancet.
403 2018;392(10152):1015-1035.
404 2.	McCambridge J, McAlaney J, Rowe R. Adult consequences of late adolescent alcohol consumption: a
405 systematic review of cohort studies. PLoS medicine. 2011;8(2):e1000413.
406 3.	World Health Organization. Global status report on alcohol and health 2018,. 2019.
407 [https://www.who.int/publications/i/item/9789241565639.](https://www.who.int/publications/i/item/9789241565639) Accessed 15 August 2023.
408 4.	Patton GC, Sawyer SM, Santelli JS, et al. Our future: a Lancet commission on adolescent health and
409 wellbeing. The Lancet. 2016;387(10036):2423-2478.
410 5.	Toumbourou JW. Implementing Communities That Care in Australia: a community mobilisation
411 approach to crime prevention. Trends and issues in crime and criminal justice. 1999(122):1 -6.
412 6.	Toumbourou JW, Rowland B, Williams J, Smith R, Patton G. Community intervention to prevent
413 adolescent health behavior problems: evaluation of Communities that Care in Australia. . Health Psychology.
414 2019.
415 7.	Communities That Care. 5 Phases of CTC. 2023. [https://www.communitiesthatcare.org.au/5-phases-ctc.](https://www.communitiesthatcare.org.au/5-phases-ctc)
416 Accessed 10 November 2023.
417 8.	Fagan AA, Hawkins JD, Catalano RF. Communities that care: Building community engagement and
418 capacity to prevent youth behavior problems: Oxford University Press, USA; 2018.
419 9.	McBride NT, Farringdon FH, Kennedy CA. Research to practice—formal dissemination of the School
420 Health and Alcohol Harm Reduction Project (SHAHRP) in Australia. Drug and Alcohol Review. 2007;26(6):665 -
421 672.
422 10.	Sanders MR, Kirby JN, Tellegen CL, Day JJ. The Triple P-Positive Parenting Program: A systematic
423 review and meta-analysis of a multi-level system of parenting support. Clinical psychology review.
424 2014;34(4):337-357.
425 11.	Hawkins J, Oesterle S, Brown EC, et al. Results of a type 2 translational research trial to prevent
426 adolescent drug use and delinquency: A test of communities that care. Archives of Pediatrics & Adolescent
427 Medicine. 2009;163(9):789-798.
428 12.	Hawkins J, Oesterle S, Brown EC, et al. Sustained decreases in risk exposure and youth problem
429 behaviors after installation of the communities that care prevention system in a randomized trial. Archives of
430 Pediatrics & Adolescent Medicine. 2012;166(2):141-148.
431 13.	Drummond MF, Sculpher MJ, Claxton K, Stoddart GL, Torrance GW. Methods for the economic
432 evaluation of health care programmes: Oxford university press; 2015.
433 14.	Population by Age and Sex, Australia 2006 [Internet]. 2007 [cited February 2017].
434 15.	Australian Bureau Statistics. 3235.0 Population by Age and Sex, Regions of Australia Canberra:
435 Australian	Bureau	of	Statistics	2012	[updated	Februaury	2017.
436 2012:[[http://www.abs.gov.au/AUSSTATS/abs@.nsf/DetailsPage/3235.02011?OpenDocument .](http://www.abs.gov.au/AUSSTATS/abs%40.nsf/DetailsPage/3235.02011?OpenDocument) Accessed	10
437 January 2021.
438 16.	Australian Bureau of Statistics. Estimated Resident Population : ERP by LGA (ASGS 2013), Age and
439 Sex, 2001 to 2013. 2013. Data extracted on 13 Apr 2017 06:44 UTC (GMT) from ABS.Stat © Commonwealth of
440 Australia. Creative Commons Attribution 2.5 Australia (<https://creativecommons.org/licenses/by/2.5/au>).
441 Accessed April 2017.
442 17.	Kelly AB, Evans‐Whipp TJ, Smith R, et al. A longitudinal study of the association of adolescent polydrug
443 use, alcohol use and high school non‐completion. Addiction 2015;110:627-635.
444 18.	Degenhardt L, O'Loughlin C, Swift W, et al. The persistence of adolescent binge drinking into adulthood:
445 findings from a 15-year prospective cohort study. BMJ open. 2013;3(8):e003015.
446 19.	Kuklinski MR, Briney JS, Hawkins JD, Catalano RF. Cost-benefit analysis of communities that care
447 outcomes at eighth grade. Prevention Science. 2012;13(2):150 -161.
448 20.	Australian Institute of healtha nd Welfare. 2010 National Drug Strategy Household Survey report.
449 Australian Institute of Health and Welfare; 2011.
450 21.	Health AIo, Welfare. National Drug Strategy Household Survey detailed report: 2013. Canberra: AIHW;
451 2014.
452 22.	Laslett A, Catalano P, Chikritzhs T, et al. The range and magnitude of alcohol’s harm to others. Fit zroy:
453 AER Centre for Alcohol Policy Research, Turning Point Alcohol and Drug Centre, Eastern Health; 2010.
454 23.	Turning Point. AODstats - Victoria State.	Retrieved Februaury, 2017, from
455 <http://aodstats.org.au/VicState/> 2017. [http://aodstats.org.au/VicState/.](http://aodstats.org.au/VicState/) Accessed Februaury, 2017.
456 24.	Watts JJ, Abimanyi-Ochom J, Sanders KM. Osteoporosis costing all Australian: a new burden of disease
457 analysis-2012 to 2022. 2013.
458 25.	Smith RG, Jorna P, Sweeney J, Fuller G. Counting the costs of crime in Australia: A 2011 estimate. AIC
459 reports Research and Public Policy series. 2014:xvii.
460 26.	Manning M, Smith C, Mazerolle P. The societal costs of alcohol misuse in Australia. Trends and issues
461 in crime and criminal justice. 2013(454):1-6.
462 27.	Australian Bureau of Statistics. Recorded Crime Offenders 'Offenders, Australia' [data set]. 2022.
463 <https://www.abs.gov.au/statistics/people/crime-and-justice/recorded-crime-offenders/latest-release#content>
464 Accessed 10 March 2022.
465 28.	Palk G, Davey J, Freeman J. Prevalence and characteristics of alcohol-related incidents requiring police
466 attendance. Journal of Studies on Alcohol and Drugs. 2007;68(4):575 -581.
467 29.	Collins D, Lapsley HM. The costs of tobacco, alcohol and illicit drug abuse to Australian society in
468 2004/05: Department of Health and Ageing Canberra; 2008.
469 30.	McCollister KE, French MT, Fang H. The cost of crime to society: new crime-specific estimates for
470 policy and program evaluation Drug and Alcohol Dependence. 2010;108::98 –109.
471 31.	NHMRC. Australian guidelines to reduce health risks from drinking alcohol. Canberra, ACT: National
472 Health and Medical Research Council; 2009.
473 32.	Cadilhac D, Magnus A, Cumming T, et al. The health and economic benefits of reducing disease risk
474 factors. Prepared for VicHealth by Deakin Health Economics Group, Deakin Universit y and National Stroke
475 Research Institute
476 2009.
477 33.	Vos T, Carter R, Barendregt J, et al. Assessing cost-effectiveness in prevention. The University of
478 Queensland, Brisbane, and Deakin University, Melbourne. 2010.
479 34.	Australian Institute of healtha nd Welfare. Annal rates of health inflation, 1999-00 to 2009-19. 2011
480 [http://www.aihw.gov.au/publication-detail/?id=10737420435.](http://www.aihw.gov.au/publication-detail/?id=10737420435) Accessed February 20 2022.
481 35.	Reserve	Bank	of	Australia.	Infaltion	calculator.	Reserve	Bank	of	Australia.
482 [https://www.rba.gov.au/calculator/annualDecimal.html.](https://www.rba.gov.au/calculator/annualDecimal.html) Accessed April 26, 2020 2022.
483 36.	Miller TR, Levy DT, Spicer RS, Taylor DM. Societal costs of underage drinking. Journal of studies on
484 alcohol. 2006;67(4):519-528.
485 37.	Communities That Care. A guide to Australian Prevention Strategies. Victoria: Communities That Care
486 Ltda. 2012.
487 38.	Charter O. Ottawa Charter for health promotion. First international conference on health promotion.
488 1986;21:17-21.
489 39.	Perry CL, Williams CL, Veblen-Mortenson S, et al. Project northland: Outcomes from a communitywide
490 alcohol use prevention program during early adolescence. American Journal of Public Health. 199 6;86(7):956 -
491 965.
492 40.	Toumbourou JW, Rowland B, Matin G-M, Patton G, Williams J. Student survey trends in reported
493 alcohol use and influencing factors in Australia. Drug and Alcohol Review. 2018.
494 41.	Australian Bureau of Statistics. 4364.0.55.001 - National Health Survey: First Results, 2014-2015.
495 Canberra:	Australian	Bureau	of	Statistics	2015	[updated	August	2019.	December
496 2015:[[https://www.abs.gov.au/ausstats/abs@.nsf/Lookup/by%20Subject/4364.0.55.001~2014-](https://www.abs.gov.au/ausstats/abs%40.nsf/Lookup/by%20Subject/4364.0.55.001~2014-15~Main%20Features~Alcohol%20consumption~25)
497 [15~Main%20Features~Alcohol%20consumption~25.](https://www.abs.gov.au/ausstats/abs%40.nsf/Lookup/by%20Subject/4364.0.55.001~2014-15~Main%20Features~Alcohol%20consumption~25) Accessed 10 January 2021.
498 42.	Rowland B, Kelly AB, Mohebbi M, et al. Evaluation of communities that care—Effects on municipal
499 youth crime rates in Victoria, Australia: 2010–2019. Prevention science. 2022:1-12.

500

Response to Reviewers

**Manuscript: PONE-D-24-08327- Response to Editorial and Reviewer comments**

Dear Editor,

Thank you for reviewing our original manuscript and for the opportunity to resubmit our paper. Our responses to the editor and reviewers are in point format in the table below. We hope our answers are more precise and clarify the questions/comments raised.

Kind regards,

Julie Abimanyi-Ochom

Response to reviewer comments

| **Comment reference number** | **Comments** | **Response** |
| --- | --- | --- |
| Editor/ journal requirement | |  |
| 1 | When submitting your revision, we need you to address these additional requirements.  Please ensure that your manuscript meets PLOS ONE's style requirements, including those for file naming. The PLOS ONE style templates can be found at [https://journals.plos.org/plosone/s/file?id=wjVg/PLOSOne_formatting](https://aus01.safelinks.protection.outlook.com/?url=https%3A%2F%2Fjournals.plos.org%2Fplosone%2Fs%2Ffile%3Fid%3DwjVg%2FPLOSOne_formatting_sample_main_body.pdf&data=05%7C02%7Cshalika.b%40deakin.edu.au%7C3da19c0245194b64472e08dc8a7b9e38%7Cd02378ec168846d585401c28b5f470f6%7C0%7C0%7C638537510746710129%7CUnknown%7CTWFpbGZsb3d8eyJWIjoiMC4wLjAwMDAiLCJQIjoiV2luMzIiLCJBTiI6Ik1haWwiLCJXVCI6Mn0%3D%7C0%7C%7C%7C&sdata=jWBhcww72k9%2B4%2F4zdnTXW8CDK6dMwqhvnkBZeP4Tklg%3D&reserved=0)  [_sample_main_body.pdf](https://aus01.safelinks.protection.outlook.com/?url=https%3A%2F%2Fjournals.plos.org%2Fplosone%2Fs%2Ffile%3Fid%3DwjVg%2FPLOSOne_formatting_sample_main_body.pdf&data=05%7C02%7Cshalika.b%40deakin.edu.au%7C3da19c0245194b64472e08dc8a7b9e38%7Cd02378ec168846d585401c28b5f470f6%7C0%7C0%7C638537510746710129%7CUnknown%7CTWFpbGZsb3d8eyJWIjoiMC4wLjAwMDAiLCJQIjoiV2luMzIiLCJBTiI6Ik1haWwiLCJXVCI6Mn0%3D%7C0%7C%7C%7C&sdata=jWBhcww72k9%2B4%2F4zdnTXW8CDK6dMwqhvnkBZeP4Tklg%3D&reserved=0) and [https://journals.plos.org/plosone/s/file?id=ba62/PLOSOne_formatting](https://aus01.safelinks.protection.outlook.com/?url=https%3A%2F%2Fjournals.plos.org%2Fplosone%2Fs%2Ffile%3Fid%3Dba62%2FPLOSOne_formatting_sample_title_authors_affiliations.pdf&data=05%7C02%7Cshalika.b%40deakin.edu.au%7C3da19c0245194b64472e08dc8a7b9e38%7Cd02378ec168846d585401c28b5f470f6%7C0%7C0%7C638537510746723894%7CUnknown%7CTWFpbGZsb3d8eyJWIjoiMC4wLjAwMDAiLCJQIjoiV2luMzIiLCJBTiI6Ik1haWwiLCJXVCI6Mn0%3D%7C0%7C%7C%7C&sdata=UHRvThpNXUPIPH5miAfQt2AaEirKSvUoOIkQdVL%2FK8s%3D&reserved=0)  [_sample_title_authors_affiliations.pdf](https://aus01.safelinks.protection.outlook.com/?url=https%3A%2F%2Fjournals.plos.org%2Fplosone%2Fs%2Ffile%3Fid%3Dba62%2FPLOSOne_formatting_sample_title_authors_affiliations.pdf&data=05%7C02%7Cshalika.b%40deakin.edu.au%7C3da19c0245194b64472e08dc8a7b9e38%7Cd02378ec168846d585401c28b5f470f6%7C0%7C0%7C638537510746723894%7CUnknown%7CTWFpbGZsb3d8eyJWIjoiMC4wLjAwMDAiLCJQIjoiV2luMzIiLCJBTiI6Ik1haWwiLCJXVCI6Mn0%3D%7C0%7C%7C%7C&sdata=UHRvThpNXUPIPH5miAfQt2AaEirKSvUoOIkQdVL%2FK8s%3D&reserved=0). | Thank you for the positive feedback. We have reviewed and applied PLOS ONE’s style requirements to our manuscript. |
| 2 | Thank you for stating the following financial disclosure:  [Bosco Rowland, John W Toumbourou and Rob Carter received funding from The National Health and Medical Research Council (APP1087781) and Australian Research Council (LP100200755).]. Please state what role the funders took in the study. If the funders had no role, please state: ""The funders had no role in study design, | Thank you for highlighting this.  Please change the **financial disclosure** in the online submission form as  “*The funders had no role in study design, data collection and analysis, decision to publish, or preparation of the manuscript*” |

| **Comment**  **reference number** | **Comments** | **Response** |
| --- | --- | --- |
|  | data collection and analysis, decision to publish, or preparation of the manuscript.""  If this statement is not correct you must amend it as needed.  Please include this amended Role of Funder statement in your cover letter; we will change the online submission form on your behalf. | This has been added to the cover letter. |
| 3 | Thank you for stating the following in the Acknowledgments Section of your manuscript:  [Bosco Rowland, John W Toumbourou and Rob Carter received funding from the National Health and Medical Research Council (APP1087781) and Australian Research Council (LP100200755).] We note that you have provided funding information that is not currently declared in your Funding Statement. However, funding information should not appear in the Acknowledgments section or other areas of your manuscript. We will only publish funding information present in the Funding Statement section of the online submission form.  Please remove any funding-related text from the manuscript and let us know how you would like to update your Funding Statement.  Currently, your Funding Statement reads as follows:  [Bosco Rowland, John W Toumbourou and Rob Carter received funding from The National Health and Medical Research Council (APP1087781) and Australian Research Council (LP100200755).]. Please include your amended statements within your cover letter; we will change the online submission form on your behalf. | Thank you for highlighting this.  Please change/ add the **funding information** in the online submission form as  “*Bosco Rowland, John W Toumbourou and Rob Carter received funding from The National Health and Medical Research Council (APP1087781) and Australian Research Council (LP100200755)*”  Manuscript changes: Page 23, lines 387-389 We removed the funding acknowledgment in the manuscript. |
| 4 | Thank you for stating the following in the Competing Interests section:  [The following authors served in the not-for-profit company  Communities That Care Ltd that was evaluated in this paper: | Thank you for highlighting this. This information has been added to the cover letter. |

| **Comment**  **reference number** | **Comments** | **Response** |
| --- | --- | --- |
|  | Rowland (current Chief Executive Officer, CEO) and Toumbourou (Director and ex-CEO). Conflict of interest management strategies were approved by their Deakin University employer.].  Please confirm that this does not alter your adherence to all PLOS ONE policies on sharing data and materials, by including the following statement: ""This does not alter our adherence to PLOS ONE policies on sharing data and materials.” (as detailed online in our guide for authors <http://journals.plos.org/plosone/s/competing-> interests). If there are restrictions on sharing of data and/or materials, please state these. Please note that we cannot proceed with consideration of your article until this information has been declared.  Please include your updated Competing Interests statement in your cover letter; we will change the online submission form on your behalf. |  |
| 5 | We note that you have indicated that there are restrictions to data sharing for this study. PLOS only allows data to be available upon request if there are legal or ethical restrictions on sharing data publicly. For more information on unacceptable data access restrictions, please see <http://journals.plos.org/plosone/s/data-> availability#loc-unacceptable-data-access-restrictions.  Before we proceed with your manuscript, please address the following prompts:  a) If there are ethical or legal restrictions on sharing a de-identified data set, please explain them in detail (e.g., data contain potentially identifying or sensitive patient information, data are owned by a third- party organization, etc.) and who has imposed them (e.g., a  Research Ethics Committee or Institutional Review Board, etc.). Please also provide contact information for a data access committee, | a) The study did not use de-identified individual level data. However, the Excel model/s used identified community-level data, which cannot be shared for ethical reasons. All other model input data have been provided within the manuscript (Tables 1 and 2). |

| **Comment**  **reference number** | **Comments** | **Response** |
| --- | --- | --- |
|  | ethics committee, or other institutional body to which data requests may be sent.  b) If there are no restrictions, please upload the minimal anonymized data set necessary to replicate your study findings to a stable, public repository and provide us with the relevant URLs, DOIs, or accession numbers. For a list of recommended repositories, please see  https://journals.plos.org/plosone/s/recommended-repositories. You also have the option of uploading the data as Supporting Information files, but we would recommend depositing data directly to a data repository if possible.  We will update your Data Availability statement on your behalf to reflect the information you provide. | b) Researchers can replicate the model for the desired populations using the information in Tables 1, Table 2, and the methods section. We believe that given the model data input information, the results of our study can be replicated for a desired population of interest.  “All data relevant to the study are included in the article”. |
| 6 | In the online submission form, you indicated that [The benefit-cost model data includes multiple excel files with spreadsheets and are stored in the University secured data storage. Data can be provided on request from authors.].  All PLOS journals now require all data underlying the findings described in their manuscript to be freely available to other researchers, either 1. In a public repository, 2. Within the manuscript itself, or 3. Uploaded as supplementary information.  This policy applies to all data except where public deposition would breach compliance with the protocol approved by your research ethics board. If your data cannot be made publicly available for ethical or legal reasons (e.g., public availability would compromise patient privacy), please explain your reasons on resubmission and your exemption request will be escalated for approval. | Thank you for highlighting this.  Our intent was to mention that this study did not have an individual dataset, and the modelling exercise used multiple Excel spreadsheets. As mentioned above, these spreadsheets comprise identified communities that cannot be shared.  For clarification, we have changed this statement to  “All data relevant to the study are included in the article”  All data relevant to the study are included within the manuscript, and researchers can replicate the model for the desired populations using the information in Tables 1, Table 2, and the methods section. |

| **Comment**  **reference number** | **Comments** | **Response** |
| --- | --- | --- |
| 7 | Please include captions for your Supporting Information files at the end of your manuscript, and update any in-text citations to match accordingly. Please see our Supporting Information guidelines for more information: <http://journals.plos.org/plosone/s/supporting-> information. | The captions for the Supporting information have been added to Page 23, lines 395-397  “Supporting Information”  Supporting information S1: Duration of the Australian CTC implementation”  Supporting information S1: Example anonymised Australian CTC community cost template |
| Reviewer |  |  |
| R1 | Is the manuscript technically sound, and do the data support the conclusions?  The manuscript must describe a technically sound piece of scientific research with data that supports the conclusions. Experiments must have been conducted rigorously, with appropriate controls, replication, and sample sizes. The conclusions must be drawn appropriately based on the data presented.  Reviewer #1: No | Thank you for your feedback.  This is a health economic modelling study. The method of the manuscript describes technically sound detailed information relevant to the modelling study such as the modelling reference period, target population, perspective and model inputs.  Given the nature of this study, therefore, the manuscript does not have information relevant to experimental research study (for example, controls, sample size etc).  The study conclusion is coherent with the study aim, method, results, and findings in the discussion. We believe the conclusion was drawn appropriately from the results and findings.  Additionally, the study methodology, results and conclusion are consistent with other CTC cost benefit studies.  Kuklinski MR, Briney JS, Hawkins JD, Catalano RF. Cost-benefit analysis of communities that care outcomes at eighth grade. |

| **Comment**  **reference number** | **Comments** | **Response** |
| --- | --- | --- |
|  |  | Prevention Science. 2012;13(2):150-161 <https://www.ncbi.nlm.nih.gov/pmc/articles/PMC3305832/> |
| R2 | Has the statistical analysis been performed appropriately and rigorously?  Reviewer #1: I Don't Know | No statistical analysis conducted for this study. The cost benefit modelling has been performed appropriately and is consistent with previously published CTC cost benefit.  Analysis details are on page 8 to 10. |
| R3 | Have the authors made all data underlying the findings in their manuscript fully available?  The [PLOS Data policy](https://aus01.safelinks.protection.outlook.com/?url=http%3A%2F%2Fwww.plosone.org%2Fstatic%2Fpolicies.action%23sharing&data=05%7C02%7Cs.wanniarachchigedona%40deakin.edu.au%7C747ea2cef8724fc9167a08dc8a798ca4%7Cd02378ec168846d585401c28b5f470f6%7C0%7C0%7C638537501892066403%7CUnknown%7CTWFpbGZsb3d8eyJWIjoiMC4wLjAwMDAiLCJQIjoiV2luMzIiLCJBTiI6Ik1haWwiLCJXVCI6Mn0%3D%7C0%7C%7C%7C&sdata=9gCBJtjnylACBhQk6%2F8m72j0ixXbGuxKp9Fl57hQXo4%3D&reserved=0) requires authors to make all data underlying the findings described in their manuscript fully available without restriction, with rare exception (please refer to the Data Availability Statement in the manuscript PDF file). The data should be provided as part of the manuscript or its supporting information, or deposited to a public repository. For example, in addition to summary statistics, the data points behind means, medians and variance measures should be available. If there are restrictions on publicly sharing data—e.g. participant privacy or use of data from a third party— those must be specified.  Reviewer #1: Yes |  |

| **Comment**  **reference number** | **Comments** | **Response** |
| --- | --- | --- |
| R4 | Is the manuscript presented in an intelligible fashion and written in standard English?  PLOS ONE does not copyedit accepted manuscripts, so the language in submitted articles must be clear, correct, and unambiguous. Any typographical or grammatical errors should be corrected at revision, so please note any specific errors here.  Reviewer #1: Yes |  |
| R5 | Australian Communities that Care Intervention: B-C analysis of a community-based youth alcohol prevention initiative.  As the authors note adolescent and youth alcohol drinking is a public health concern. And evidence on what works and is cost-effective is valuable.  However despite being nicely written the underlying logic is not convincing, and in particular whether the trial outcomes that inform this B-C analysis are suitable to answer the research question. | Thank you for your feedback.  The CTC trial showed positive outcomes in adolescent alcohol and drug use, and related antisocial behaviors, less favorable parent attitudes toward alcohol and drug use, and reduced community availability of alcohol. Given these positive outcomes, the benefit-cost model was developed to explore the economic benefits to society by implementing the CTC trial based on the positive outcomes in adolescent alcohol use.  Therefore, the model used the CTC outcome of reduction in alcohol use to explore the benefit through avoided alcohol related events (costs and/or benefits) in terms of alcohol- related hospital admissions, emergency department visits, ambulance attendances, alcohol treatment programs, deaths, and crime because of the CTC trial. |

| **Comment**  **reference number** | **Comments** | **Response** |
| --- | --- | --- |
| R6 | A Cost - Benefit analysis relies entirely on the interpretation of reported outcomes, in this case of a CTC intervention in 5 localities. As such it would be useful to restate the key outcomes being draw on and what they mean. As I understand the outcomes from the Toumbourou et al 2019 paper as reported, the key question related to alcohol assumption was about number of drinks a year 8 student had even consumed - defined as more than a few sips of an alcoholic beverage ever – dichotomised into ever vs never. It is not self-evident that this would be a useful descriptor of alcohol consumption from which to model impacts of alcohol consumption on ED attendance, criminal behaviour etc – which might rather have been related more to intoxication. If this is a mis-interpretation please describe more clearly the trial data on which the modelling is based. But if correct, greater attention as to why this outcome is considered sound for assessing the impacts of alcohol needs to be made. The  B-C analysis rests on this. | Thank you for this comment and for further clarity we added a new paragraph (Page 5, lines 117-123) explaining the CTC outcomes used and their meanings in this benefit-cost analysis.  The benefit-cost model predicts the cost and benefits in youth population using the reduction in alcohol use due to CTC trial. Firstly, the reduction in alcohol use was used to estimate the alcohol consuming population. Secondly, based on the rate of risky alcohol consumption in this population, we estimated the population that have risky alcohol consumption. We estimated avoided health and non-health consequences due to risky alcohol consumption, similar to other CTC studies.  Page 5, line 115-123  “The benefit-cost model estimated the cost of the intervention and the benefits in the youth population based on the reduction in alcohol use due to the CTC trial. Firstly, the reduction in alcohol use measured in the trial was used to estimate the reduction in the alcohol consuming population due to the intervention for the intervention population. Secondly, we estimated the population that have risky alcohol consumption within the alcohol consuming intervention population. Lastly, avoided consequences in health-related events (alcohol-related hospital admissions, emergency department visits, ambulance attendances, alcohol treatment programs), and in non-health events (deaths, and crime) due to risky alcohol consumption were estimated and costed to give the benefit from the CTC intervention”. |

| **Comment**  **reference number** | **Comments** | **Response** |
| --- | --- | --- |
| R6 | In particular, given the vast majority of benefits were from a modelled crime reduction, evidence to support the plausibility of the assumptions here are crucial. For example does a PAF for alcohol related youth crime, necessarily translate into what would be avoided if alcohol consumption was reduced – is the PAF is based on an association or a causal link. Further in terms of evidence from this study, perhaps explain why a 1%pa reduced likely of ever having a drink in year 8 in a community is linked to the number of adolescents who might get intoxicated and perhaps more likely to participate in crime. There seems to be a few logic steps missing. | The model did not use direct results from the Toumbourou study. The model used the interpolated estimates (of the Toumbourou study results) for an average absolute reduction in alcohol use (1.01% per year) due to the CTC trial (explained in lines 140-145). A citation was added to confirm our attributed crime reduction is consistent with that measured in an evaluation of reductions in police-reported crime due to the Australian CTC implementation.  We added a new paragraph (Page 5, lines 117-123) explaining the CTC outcomes used and their meanings in this benefit-cost analysis. In this case, all included crime only related to alcohol related/attributable crime from existing literature as shown in Table 1. We also noted that the estimated crime reduction aligns with that measured in evaluations of CTC. |
| R5 | The survey on which the CTC evaluation was based did include questions on anti-social behaviour including involvement in criminal activity – but it appears this wasn’t used in the C-B analysis. It is not clear why not?  Finally, ensuring the intervention is fully costed is clearly important. I could not locate the details on how the costing was done.  Specifically what was the source of the costing data, do costs reflect | The study aim was to estimate the avoided health-related events (alcohol-related hospital admissions, emergency department visits, ambulance attendances, alcohol treatment programs), and in non-health events (deaths, and crime) due to risky alcohol consumption. Anti-social behaviour was not considered in this model.  We’ve added a footnote for table 3 about the intervention cost. The paper did not detail the intervention cost in the method since the intervention costing was complex and included costing the intervention cost categories for each community coalition. This would detract from the aim of this  manuscript to demonstrate how the costs and benefits compared. |

| **Comment**  **reference number** | **Comments** | **Response** |
| --- | --- | --- |
|  | a full cost, for example if office space were made available at below- market rate – how was that treated? It is unclear what is covered under salary costs – does it include wage on-costs and salary overheads (eg for HR, financial services, IT other agency costs). | The costing method used was appropriate: most of the  included costs are based on actual expenses – for example the student survey cost, training costs, salary allocation costs |
|  |  | – based on one’s market rate renumeration for their level of |
|  |  | expertise, rental space costs was based on the room hiring |
|  |  | cost paid when the room was needed, so rental on-costs |
|  |  | were not an issue, volunteer’s time was costed at the |
|  |  | opportunity cost of time based on the market rate per hour. |
|  |  | Inclusion of the costing details within the methods will make |
|  |  | the paper complex so this has not been included within the |
|  |  | paper. However, an example CTC community costing |
|  |  | complete has been included in the Supporting information |
|  |  | section in addition to the general costing method included in |
|  |  | the manuscript. |
|  |  | S2 Fig: Example anonymised Australian CTC community cost template” |
