# Supplementary material for: Australian Communities That Care (CTC) intervention: Benefit-cost analysis of a community-based youth alcohol prevention initiative
Source: PLoS One. 2024 Nov 27;19(11):e0314153. doi: 10.1371/journal.pone.0314153 (PMC11602060; doi:10.1371/journal.pone.0314153)
Supplement: Attachment — Submitted filename: Reviewer comments - 26072024.docx [file pone.0314153.s003.docx]

**Manuscript: PONE-D-24-08327- Response to Editorial and Reviewer comments**

Dear Editor,

Thank you for reviewing our original manuscript and for the opportunity to resubmit our paper. Our responses to the editor and reviewers are in point format in the table below. We hope our answers are more precise and clarify the questions/comments raised.

Kind regards,

Julie Abimanyi-Ochom

Response to reviewer comments

| **Comment reference number** | **Comments** | **Response** |
| --- | --- | --- |
| Editor/ journal requirement | |  |
| 1 | When submitting your revision, we need you to address these additional requirements.  Please ensure that your manuscript meets PLOS ONE's style requirements, including those for file naming. The PLOS ONE style templates can be found at  [https://journals.plos.org/plosone/s/file?id=wjVg/PLOSOne_formatting_sample_main_body.pdf](https://aus01.safelinks.protection.outlook.com/?url=https%3A%2F%2Fjournals.plos.org%2Fplosone%2Fs%2Ffile%3Fid%3DwjVg%2FPLOSOne_formatting_sample_main_body.pdf&data=05%7C02%7Cshalika.b%40deakin.edu.au%7C3da19c0245194b64472e08dc8a7b9e38%7Cd02378ec168846d585401c28b5f470f6%7C0%7C0%7C638537510746710129%7CUnknown%7CTWFpbGZsb3d8eyJWIjoiMC4wLjAwMDAiLCJQIjoiV2luMzIiLCJBTiI6Ik1haWwiLCJXVCI6Mn0%3D%7C0%7C%7C%7C&sdata=jWBhcww72k9%2B4%2F4zdnTXW8CDK6dMwqhvnkBZeP4Tklg%3D&reserved=0)  and  [https://journals.plos.org/plosone/s/file?id=ba62/PLOSOne_formatting_sample_title_authors_affiliations.pdf](https://aus01.safelinks.protection.outlook.com/?url=https%3A%2F%2Fjournals.plos.org%2Fplosone%2Fs%2Ffile%3Fid%3Dba62%2FPLOSOne_formatting_sample_title_authors_affiliations.pdf&data=05%7C02%7Cshalika.b%40deakin.edu.au%7C3da19c0245194b64472e08dc8a7b9e38%7Cd02378ec168846d585401c28b5f470f6%7C0%7C0%7C638537510746723894%7CUnknown%7CTWFpbGZsb3d8eyJWIjoiMC4wLjAwMDAiLCJQIjoiV2luMzIiLCJBTiI6Ik1haWwiLCJXVCI6Mn0%3D%7C0%7C%7C%7C&sdata=UHRvThpNXUPIPH5miAfQt2AaEirKSvUoOIkQdVL%2FK8s%3D&reserved=0). | Thank you for the positive feedback. We have reviewed and applied PLOS ONE’s style requirements to our manuscript. |
| 2 | Thank you for stating the following financial disclosure:  [Bosco Rowland, John W Toumbourou and Rob Carter received funding from The National Health and Medical Research Council (APP1087781) and Australian Research Council (LP100200755).].  Please state what role the funders took in the study. If the funders had no role, please state: ""The funders had no role in study design, data collection and analysis, decision to publish, or preparation of the manuscript.""  If this statement is not correct you must amend it as needed.  Please include this amended Role of Funder statement in your cover letter; we will change the online submission form on your behalf. | Thank you for highlighting this.  Please change the **financial disclosure** in the online submission form as  “*The funders had no role in study design, data collection and analysis, decision to publish, or preparation of the manuscript*”  This has been added to the cover letter. |
| 3 | Thank you for stating the following in the Acknowledgments Section of your manuscript:  [Bosco Rowland, John W Toumbourou and Rob Carter received funding from the National Health and Medical Research Council (APP1087781) and Australian Research Council (LP100200755).]  We note that you have provided funding information that is not currently declared in your Funding Statement. However, funding information should not appear in the Acknowledgments section or other areas of your manuscript. We will only publish funding information present in the Funding Statement section of the online submission form.  Please remove any funding-related text from the manuscript and let us know how you would like to update your Funding Statement. Currently, your Funding Statement reads as follows:  [Bosco Rowland, John W Toumbourou and Rob Carter received funding from The National Health and Medical Research Council (APP1087781) and Australian Research Council (LP100200755).].  Please include your amended statements within your cover letter; we will change the online submission form on your behalf. | Thank you for highlighting this.  Please change/ add the **funding information** in the online submission form as  “*Bosco Rowland, John W Toumbourou and Rob Carter received funding from The National Health and Medical Research Council (APP1087781) and Australian Research Council (LP100200755)*”  Manuscript changes: Page 23, lines 387-389  We removed the funding acknowledgment in the manuscript. |
| 4 | Thank you for stating the following in the Competing Interests section:  [The following authors served in the not-for-profit company Communities That Care Ltd that was evaluated in this paper:  Rowland (current Chief Executive Officer, CEO) and Toumbourou (Director and ex-CEO). Conflict of interest management strategies were approved by their Deakin University employer.].  Please confirm that this does not alter your adherence to all PLOS ONE policies on sharing data and materials, by including the following statement: ""This does not alter our adherence to PLOS ONE policies on sharing data and materials.” (as detailed online in our guide for authors http://journals.plos.org/plosone/s/competing-interests). If there are restrictions on sharing of data and/or materials, please state these. Please note that we cannot proceed with consideration of your article until this information has been declared.  Please include your updated Competing Interests statement in your cover letter; we will change the online submission form on your behalf. | Thank you for highlighting this. This information has been added to the cover letter. |
| 5 | We note that you have indicated that there are restrictions to data sharing for this study. PLOS only allows data to be available upon request if there are legal or ethical restrictions on sharing data publicly. For more information on unacceptable data access restrictions, please see http://journals.plos.org/plosone/s/data-availability#loc-unacceptable-data-access-restrictions.  Before we proceed with your manuscript, please address the following prompts:  a) If there are ethical or legal restrictions on sharing a de-identified data set, please explain them in detail (e.g., data contain potentially identifying or sensitive patient information, data are owned by a third-party organization, etc.) and who has imposed them (e.g., a Research Ethics Committee or Institutional Review Board, etc.). Please also provide contact information for a data access committee, ethics committee, or other institutional body to which data requests may be sent.  b) If there are no restrictions, please upload the minimal anonymized data set necessary to replicate your study findings to a stable, public repository and provide us with the relevant URLs, DOIs, or accession numbers. For a list of recommended repositories, please see  https://journals.plos.org/plosone/s/recommended-repositories. You also have the option of uploading the data as Supporting Information files, but we would recommend depositing data directly to a data repository if possible.  We will update your Data Availability statement on your behalf to reflect the information you provide. | a) The study did not use de-identified individual level data. However, the Excel model/s used identified community-level data, which cannot be shared for ethical reasons. All other model input data have been provided within the manuscript (Tables 1 and 2).  b) Researchers can replicate the model for the desired populations using the information in Tables 1, Table 2, and the methods section. We believe that given the model data input information, the results of our study can be replicated for a desired population of interest.  “All data relevant to the study are included in the article”. |
| 6 | In the online submission form, you indicated that [The benefit-cost model data includes multiple excel files with spreadsheets and are stored in the University secured data storage. Data can be provided on request from authors.].  All PLOS journals now require all data underlying the findings described in their manuscript to be freely available to other researchers, either 1. In a public repository, 2. Within the manuscript itself, or 3. Uploaded as supplementary information.  This policy applies to all data except where public deposition would breach compliance with the protocol approved by your research ethics board. If your data cannot be made publicly available for ethical or legal reasons (e.g., public availability would compromise patient privacy), please explain your reasons on resubmission and your exemption request will be escalated for approval. | Thank you for highlighting this.  Our intent was to mention that this study did not have an individual dataset, and the modelling exercise used multiple Excel spreadsheets. As mentioned above, these spreadsheets comprise identified communities that cannot be shared.  For clarification, we have changed this statement to  “All data relevant to the study are included in the article”  All data relevant to the study are included within the manuscript, and researchers can replicate the model for the desired populations using the information in Tables 1, Table 2, and the methods section. |
| 7 | Please include captions for your Supporting Information files at the end of your manuscript, and update any in-text citations to match accordingly. Please see our Supporting Information guidelines for more information: http://journals.plos.org/plosone/s/supporting-information. | The captions for the Supporting information have been added to Page 23, lines 395-397  “Supporting Information”  Supporting information S1: Duration of the Australian CTC implementation”  Supporting information S1: Example anonymised Australian CTC community cost template |
| Reviewer |  |  |
| R1 | Is the manuscript technically sound, and do the data support the conclusions?  The manuscript must describe a technically sound piece of scientific research with data that supports the conclusions. Experiments must have been conducted rigorously, with appropriate controls, replication, and sample sizes. The conclusions must be drawn appropriately based on the data presented.  Reviewer #1: No | Thank you for your feedback.  This is a health economic modelling study. The method of the manuscript describes technically sound detailed information relevant to the modelling study such as the modelling reference period, target population, perspective and model inputs.  Given the nature of this study, therefore, the manuscript does not have information relevant to experimental research study (for example, controls, sample size etc).  The study conclusion is coherent with the study aim, method, results, and findings in the discussion. We believe the conclusion was drawn appropriately from the results and findings.  Additionally, the study methodology, results and conclusion are consistent with other CTC cost benefit studies.  Kuklinski MR, Briney JS, Hawkins JD, Catalano RF. Cost-benefit analysis of communities that care outcomes at eighth grade. Prevention Science. 2012;13(2):150-161 <https://www.ncbi.nlm.nih.gov/pmc/articles/PMC3305832/> |
| R2 | Has the statistical analysis been performed appropriately and rigorously?    Reviewer #1: I Don't Know | No statistical analysis conducted for this study. The cost benefit modelling has been performed appropriately and is consistent with previously published CTC cost benefit. Analysis details are on page 8 to 10. |
| R3 | Have the authors made all data underlying the findings in their manuscript fully available?  The [PLOS Data policy](https://aus01.safelinks.protection.outlook.com/?url=http%3A%2F%2Fwww.plosone.org%2Fstatic%2Fpolicies.action%23sharing&data=05%7C02%7Cs.wanniarachchigedona%40deakin.edu.au%7C747ea2cef8724fc9167a08dc8a798ca4%7Cd02378ec168846d585401c28b5f470f6%7C0%7C0%7C638537501892066403%7CUnknown%7CTWFpbGZsb3d8eyJWIjoiMC4wLjAwMDAiLCJQIjoiV2luMzIiLCJBTiI6Ik1haWwiLCJXVCI6Mn0%3D%7C0%7C%7C%7C&sdata=9gCBJtjnylACBhQk6%2F8m72j0ixXbGuxKp9Fl57hQXo4%3D&reserved=0) requires authors to make all data underlying the findings described in their manuscript fully available without restriction, with rare exception (please refer to the Data Availability Statement in the manuscript PDF file). The data should be provided as part of the manuscript or its supporting information, or deposited to a public repository. For example, in addition to summary statistics, the data points behind means, medians and variance measures should be available. If there are restrictions on publicly sharing data—e.g. participant privacy or use of data from a third party—those must be specified.    Reviewer #1: Yes |  |
| R4 | Is the manuscript presented in an intelligible fashion and written in standard English?  PLOS ONE does not copyedit accepted manuscripts, so the language in submitted articles must be clear, correct, and unambiguous. Any typographical or grammatical errors should be corrected at revision, so please note any specific errors here.    Reviewer #1: Yes |  |
| R5 | Australian Communities that Care Intervention: B-C analysis of a community-based youth alcohol prevention initiative. As the authors note adolescent and youth alcohol drinking is a public health concern. And evidence on what works and is cost-effective is valuable. However despite being nicely written the underlying logic is not convincing, and in particular whether the trial outcomes that inform this B-C analysis are suitable to answer the research question. | Thank you for your feedback.  The CTC trial showed positive outcomes in adolescent alcohol and drug use, and related antisocial behaviors, less favorable parent attitudes toward alcohol and drug use, and reduced community availability of alcohol. Given these positive outcomes, the benefit-cost model was developed to explore the economic benefits to society by implementing the CTC trial based on the positive outcomes in adolescent alcohol use.  Therefore, the model used the CTC outcome of reduction in alcohol use to explore the benefit through avoided alcohol related events (costs and/or benefits) in terms of alcohol-related hospital admissions, emergency department visits, ambulance attendances, alcohol treatment programs, deaths, and crime because of the CTC trial. |
| R6 | A Cost - Benefit analysis relies entirely on the interpretation of reported outcomes, in this case of a CTC intervention in 5 localities. As such it would be useful to restate the key outcomes being draw on and what they mean. As I understand the outcomes from the Toumbourou et al 2019 paper as reported, the key question related to alcohol assumption was about number of drinks a year 8 student had even consumed - defined as more than a few sips of an alcoholic beverage ever – dichotomised into ever vs never. It is not self-evident that this would be a useful descriptor of alcohol consumption from which to model impacts of alcohol consumption on ED attendance, criminal behaviour etc – which might rather have been related more to intoxication. If this is a mis-interpretation please describe more clearly the trial data on which the modelling is based. But if correct, greater attention as to why this outcome is considered sound for assessing the impacts of alcohol needs to be made. The B-C analysis rests on this. | Thank you for this comment and for further clarity we added a new paragraph (Page 5, lines 117-123) explaining the CTC outcomes used and their meanings in this benefit-cost analysis.  The benefit-cost model predicts the cost and benefits in youth population using the reduction in alcohol use due to CTC trial. Firstly, the reduction in alcohol use was used to estimate the alcohol consuming population. Secondly, based on the rate of risky alcohol consumption in this population, we estimated the population that have risky alcohol consumption. We estimated avoided health and non-health consequences due to risky alcohol consumption, similar to other CTC studies.  Page 5, line 115-123  “The benefit-cost model estimated the cost of the intervention and the benefits in the youth population based on the reduction in alcohol use due to the CTC trial. Firstly, the reduction in alcohol use measured in the trial was used to estimate the reduction in the alcohol consuming population due to the intervention for the intervention population. Secondly, we estimated the population that have risky alcohol consumption within the alcohol consuming intervention population. Lastly, avoided consequences in health-related events (alcohol-related hospital admissions, emergency department visits, ambulance attendances, alcohol treatment programs), and in non-health events (deaths, and crime) due to risky alcohol consumption were estimated and costed to give the benefit from the CTC intervention”. |
| R6 | In particular, given the vast majority of benefits were from a modelled crime reduction, evidence to support the plausibility of the assumptions here are crucial. For example does a PAF for alcohol related youth crime, necessarily translate into what would be avoided if alcohol consumption was reduced – is the PAF is based on an association or a causal link. Further in terms of evidence from this study, perhaps explain why a 1%pa reduced likely of ever having a drink in year 8 in a community is linked to the number of adolescents who might get intoxicated and perhaps more likely to participate in crime. There seems to be a few logic steps missing. | The model did not use direct results from the Toumbourou study. The model used the interpolated estimates (of the Toumbourou study results) for an average absolute reduction in alcohol use (1.01% per year) due to the CTC trial (explained in lines 140-145). A citation was added to confirm our attributed crime reduction is consistent with that measured in an evaluation of reductions in police-reported crime due to the Australian CTC implementation.  We added a new paragraph (Page 5, lines 117-123) explaining the CTC outcomes used and their meanings in this benefit-cost analysis. In this case, all included crime only related to alcohol related/attributable crime from existing literature as shown in Table 1. We also noted that the estimated crime reduction aligns with that measured in evaluations of CTC. |
| R5 | The survey on which the CTC evaluation was based did include questions on anti-social behaviour including involvement in criminal activity – but it appears this wasn’t used in the C-B analysis. It is not clear why not?  Finally, ensuring the intervention is fully costed is clearly important. I could not locate the details on how the costing was done. Specifically what was the source of the costing data, do costs reflect a full cost, for example if office space were made available at below-market rate – how was that treated? It is unclear what is covered under salary costs – does it include wage on-costs and salary overheads (eg for HR, financial services, IT other agency costs). | The study aim was to estimate the avoided health-related events (alcohol-related hospital admissions, emergency department visits, ambulance attendances, alcohol treatment programs), and in non-health events (deaths, and crime) due to risky alcohol consumption. Anti-social behaviour was not considered in this model.    We’ve added a footnote for table 3 about the intervention cost. The paper did not detail the intervention cost in the method since the intervention costing was complex and included costing the intervention cost categories for each community coalition. This would detract from the aim of this manuscript to demonstrate how the costs and benefits compared.  The costing method used was appropriate: most of the included costs are based on actual expenses – for example the student survey cost, training costs, salary allocation costs – based on one’s market rate renumeration for their level of expertise, rental space costs was based on the room hiring cost paid when the room was needed, so rental on-costs were not an issue, volunteer’s time was costed at the opportunity cost of time based on the market rate per hour. Inclusion of the costing details within the methods will make the paper complex so this has not been included within the paper. However, an example CTC community costing complete has been included in the Supporting information section in addition to the general costing method included in the manuscript.  S2 Fig: Example anonymised Australian CTC community cost template” |
