# Supplementary material for: Australian Communities That Care (CTC) intervention: Benefit-cost analysis of a community-based youth alcohol prevention initiative
Source: PLoS One. 2024 Nov 27;19(11):e0314153. doi: 10.1371/journal.pone.0314153 (PMC11602060; doi:10.1371/journal.pone.0314153)
Supplement: Attachment — Submitted filename: Response to reviewer comments.docx [file pone.0314153.s005.docx]

Response to reviewers

| **#** | **Reviewer comment** | **Response** |
| --- | --- | --- |
| 1 | While these estimates are drawn from established sources, the analysis assumes that any reduction in alcohol use will directly lead to proportional reductions in adverse events. This may oversimplify the relationship between alcohol use and health outcomes, as reductions in use do not always translate linearly to avoided event - would you be able to highlight this in the discussion/limitation section? | We appreciate this comment. As suggested, this limitation was included in the discussion section.  **The change can be found on Page 23, line 369- 371**  The model assumes that a reduction in alcohol consumption will lead to proportional decreases in adverse events. However, this relationship is not always linear, and reductions in alcohol use do not consistently result in equivalent decreases in adverse events. |
| 2 | The analysis only considers the costs from the first cycle of the intervention, despite acknowledging that there were multiple cycles during the intervention period limiting the analysis to the first cycle, the total long-term costs of maintaining or repeating the intervention are not fully captured. It may potentially underestimate the true costs of scaling or sustaining the intervention over time - however in the discussion long-term benefits are discussed in the discussion, could you clarify this? | Thank you for this comment. To clarify this, three of the four communities had one cycle; therefore, the cost was only associated with one cycle for comparability across the four communities. However, future CTCs can consider all the cycles. The evidence from this study shows that the cost per youth/year reduces over future cycles, highlighting possible economies of scale as the number of CTCs cycles increase.  **We have revised this on Page 9, line 176 -179**  Only the first cycle was considered when there were multiple cycles during the intervention period for comparability across the four communities (see S2 Example anonymised Australian CTC community cost template) (three of the four communities had only one cycle).  **Also revised on page 21, Line 317-318**  (displaying probable economies of scale as the number of CTCs cycles increase). |
| 3 | how was this assumption established? were any other variables controlled? - If you have the data to control any confounding variables, perhaps add it to the analysis, if not it may need to be acknowledged in the discussions | We appreciate this comment. This assumption was derived from the established evidence between risky alcohol consumption and alcohol related adverse events based on the NHMRC. Australian guidelines to reduce health risks from drinking alcohol. Canberra, ACT: National Health and Medical Research Council; 2009  **We have specified this on page 9, line 184 to 187**  This assumption was derived from the established evidence between risky alcohol consumption and alcohol related adverse events based on the National Health and Medical Research Council (NHMRC) guidelines. The reduction in alcohol-related adverse events were estimated for risky alcohol consumption based on the NHMRC guidelines 31. |
| 4 | Would it be beneficial to expand these definitions by providing examples of what these measure? for example I would be interested to understand what crimes were measured as being attributed to alcohol use | Thank you for this suggestion. All the adverse events are alcohol related, including crime, using data from turning point which collects data on alcohol related adverse events. **The text was updated to make this clear on pages 9-10, line 195-198.**  See response to #3 above, also line 184 to 187 |
| 5 | Perhaps an example could help non-expert readers follow? (e.g., a reduction in alcohol use might simultaneously reduce e.g: crime and hospital admissions, both of which could be considered separately | We appreciate this comment. We’ve included an example as below:  **Page 10, line 201 to 204** (e.g., when the same patient utilises the ambulance, ED, and in-hospital care for a hospital admission, these events were considered as three separate patients and then the join effect was applied to attribute those events to that one patient) |
| 6 | this approach may be limited in its ability to handle complex variables and interactions. not sure if this standard in the industry- please clarify  other confounding factors are discounted in my opinion (e.g., socioeconomic status, mental health conditions). Also, the model does not account for varying levels of alcohol consumption reduction. For example, small reductions in alcohol consumption may not result in a proportional decrease in adverse events, especially for those already engaging in harmful drinking patterns. Whereas, large reductions in consumption may have a more substantial impact, but the model does not differentiate between these effects - if you have the data for this, please include it in the analysis, if not please address this in the discussion | We understand your concern regarding confounding. In economic evaluation and modelling, it is normal practice to use Excel or Triage for health economic modelling, without controlling for confounders.  In this case, we’ve used the reduction in alcohol consumption to model averted alcohol related adverse events for risky alcohol consumers (by age group, gender and community). We acknowledge that the reduction may not be proportionate within this group and have indicated this as a limitation (please see response # 1). |
| 7 | why? This could lead to an incomplete assessment of the intervention's full value, as improvements in mental health, stress reduction, or quality of life are often central to public health initiatives and in the overall prevention of relapses in any alcohol related harms: Social Determinants of Health and Alcohol Use: Galea, S., Nandi, A., & Vlahov, D. (2004). The social epidemiology of substance use. Epidemiologic Reviews, 26(1), 36-52.  If you have the data for this, please consider including it in the analysis, if not please address this limitation in the discussion | We agree that this leads to an incomplete assessment. However, the CTC study didn’t collect quality of life data. We therefore applied a limited-social perspective. The method section has been updated to indicate this.  We say limited-social perspective (on page 5) because it does not account for quality of life and wellbeing.  This has also been considered in the limitation section (line 396). |
| 8 | I am wondering if the cost per event could vary by region? - please add a footnote to the table acknowledging this | Thank you for this query. Cost per event was comparable and not different across regions. |
| 9 | could this capture complex interactions between multiple variables. For example, changes in youth exposure to the intervention may not have the same proportional impact on different outcomes ? | We do not agree that bootstrapping in this study would adequately capture complex interactions between multiple variables. Typically, bootstrapping captures synchronous variations among all the model inputs. |
| 10 | I may not have understood this, but how was this conclusion derived if the BC analysis was only applied during the intervention period  Not clear how the long-term impact has been measured in this study | Yes, we agree – there is some misunderstanding:  We were exploring the existing evidence in the literature in the discussion. We compared our results with other studies which included both short-term and long-term impact. We have only considered short-term impact in this study, as mentioned in the method and discussion. |
| 11 | Didn’t the primary analysis only consider the 1st cycle? or did the analysis also include other cycles but did not include in the ms? | We appreciate this comment. In our analysis, we used the first cycle since all communities had the one cycle for comparability across the four communities. We acknowledge that communities can have more than one cycle and discover economies of scale, and therefore discussion about it was noteworthy.  In the method section, we have now highlighted that ¾ of the communities had only one cycle (hence, only one had two cycles).  Please see the response to comment #2. |
| 12 | I think some more limitations should be included both the Toumbourou study and the BCA rely on self-reported data to estimate alcohol use, which could lead to response bias. Self-reported measures may under-report actual consumption levels, leading to underestimated benefits of the intervention | Thank for highlighting this. We have included this as a limitation as suggested.  **This change can be found on page 23, Line 362-365**  Self-reported data on alcohol use is a limitation due to the potential for response bias. Since adolescents are generally not expected to consume alcohol, these self-reported measures may under-report actual consumption levels, thereby obscuring the true benefits of the intervention. |
| 13 | I also wonder if the inclusion of a Cost effectiveness analysis might be suggested here? perhaps as future recommendation to avoid complicating this paper? this could give a bigger picture on the overall effectiveness of CTA when compared to alternative programs without necessarily quantifying it in monetary value…  IF you do have data to do a cost effectiveness analysis, please consider adding this, if not, as suggested above, consider adding to recommendation | Thank you for this suggestion. We included this in the conclusion as below (….incorporate more robust experimental designs,…). This has been modified as follows.  **We have revised this on Page 24 to 25, line 407-411**  Future research should examine the economic benefits of prevented youth tobacco and cannabis use as well as antisocial behavior as demonstrated in the Australian CTC Trial 6. Additionally, it should utilise more robust experimental designs, including randomised control trials, and consider average absolute reductions that accounts for age, gender, length of intervention period, and long-term benefits for cost effectiveness analysis. |

We believe that the revisions have strengthened our manuscript, and we hope it meets your expectations. Thank you once again for your insightful comments.

Sincerely,

Julie Abimanyi-Ochom

Deakin Health Economics, Deakin University
